# A Comparative Genome-Wide Transcriptome Analysis of Glucocorticoid Responder and Non-Responder Primary Human Trabecular Meshwork Cells

**DOI:** 10.3390/genes13050882

**Published:** 2022-05-15

**Authors:** Kandasamy Kathirvel, Ravinarayanan Haribalaganesh, Ramasamy Krishnadas, Veerappan Muthukkaruppan, Colin E. Willoughby, Devarajan Bharanidharan, Srinivasan Senthilkumari

**Affiliations:** 1 Department of Ocular Pharmacology, Aravind Medical Research Foundation, Madurai 625020, Tamilnadu, India; k.kathirvel93@gmail.com (K.K.); haribalaganesh@gmail.com (R.H.); 2 Department of Bioinformatics, Aravind Medical Research Foundation, Madurai 625020, Tamilnadu, India; bharani@aravind.org; 3 Glaucoma Clinic, Aravind Eye Hospital, Madurai 625020, Tamilnadu, India; krishnadas@aravind.org; 4 Department of Immunology and Stem Cell Biology, Aravind Medical Research Foundation, Madurai 625020, Tamilnadu, India; muthu@aravind.org; 5 Genomic Medicine, Biomedical Sciences Research Institute, Ulster University, Newtownabbey BT37 0QB, UK; c.willoughby@ulster.ac.uk

**Keywords:** glucocorticoid-induced ocular hypertension, human perfusion-cultured anterior segment, trabecular meshwork cells, gene expression, RNA-seq, candidate genes

## Abstract

**Aim:** To investigate genes and pathways involved in differential glucocorticoid (GC) responsiveness in human trabecular meshwork (HTM) cells using RNA sequencing. **Methods:** Using paired human donor eyes, human organ-cultured anterior segment (HOCAS) was established in one eye to characterize GC responsiveness based on intra ocular pressure (IOP) change and, in the other eye, primary HTM cell culture was established. For RNA sequencing, total RNA extracted from GC-responder (GC-R) and non-responder (GC-NR) cells after dexamethasone (DEX) or ethanol (ETH) treatment for 7d was used. Differentially expressed genes (DEGs) were compared among five groups and validated. **Results:** In total, 616 and 216 genes were identified as significantly dysregulated in Group #1 and #2 (#1: ETH vs. DEX-treated GC-R; #2: ETH vs. DEX-treated GC-NR), respectively. Around 80 genes were commonly dysregulated in Group #3 (overlapping DEGs between #1 and #2), whereas 536 and 136 genes were uniquely expressed in GC-R (#4) and GC-NR HTM (#5) cells, respectively. Pathway analysis revealed that WNT signaling, drug metabolism cytochrome p450, cell adhesion, TGF-β signaling, and MAPK signaling were associated with GC responsiveness. **Conclusion:** This is the first study reporting distinct gene signatures and their associated pathways for GC-R and GC-NR HTM cells. WNT and MAPK signaling are potential therapeutic targets for the management of GC-induced glaucoma.

## 1. Introduction

Glucocorticoid (GC) therapy is the mainstay in the management of systemic and ocular autoimmune and inflammatory diseases. GC-induced ocular hypertension (GC-OHT) and glaucoma (GIG) are serious side-effects associated with the long-term use of GC therapy. GC-OHT occurs in 40% of the population (GC responders), of which 6% are likely to develop glaucoma [1]. Individuals who show GC responsiveness are also at greater risk of developing primary open-angle glaucoma (POAG) [2,3]. More than 90% of the patients with POAG exhibit GC responsiveness which can further aggravate the optic nerve damage, leading to visual field loss [1].

Alternative splicing of the glucocorticoid receptor (GR) into GRα and GRβ splice isoforms can alter the GC response in TM cells [4]. In the nucleus, GRα homo-dimers regulate the expression, either positively or negatively, of various genes that contain GC response elements (GREs). GRβ, in contrast, is unable to bind GCs and acts as a negative regulator of GRα. The alternative splicing of GR is mediated by the splicesome proteins such as SFRS9 [5] and SFRS5 [6]. The transcriptional changes responsible for the differential GC responsiveness and raised intraocular pressure (IOP) are not clearly understood. Current glaucoma treatment therapy attempts to lower IOP with medications, laser or surgical treatment. However, currently there is no specific treatment option which specifically targets the pathogenic mechanisms responsible for the GC response in the trabecular meshwork (TM) resulting in elevated IOP.

Several ‘omics’ studies have attempted to identify the global changes in the expression of genes/proteins in TM cells in response to dexamethasone (DEX) treatment using cDNA or oligonucleotide arrays [7,8,9,10,11,12,13,14]. However, the overall findings were not consistent across these studies because of differences in study methodology, including different cell types, different duration and class of steroid treatment, and the use of different microarray platforms. Microarrays are limited due to the possibility of probe cross-hybridization, low detection thresholds, and the bias selection of specific probes. Moreover, the identification of novel transcripts and splice isoforms of the annotated genes are not possible, as the probe design only includes previously identified transcripts [15]. In addition, these studies did not include the details of the GC responsiveness and IOP response of the donor eyes from which TM cells were isolated. Therefore, it is difficult to determine if the changes in gene expression were directly or indirectly associated with GC-OHT pathogenesis or only reflected the global effect of GCs on the TM. In a bovine study, the unique expression of genes and pathways was documented for glucocorticoid responder (GC-R; raised IOP with steroid treatment) and glucocorticoid non-responder (GC-NR; no raised IOP with steroid treatment) TM cells [16]. In this study, a perfusion-cultured bovine anterior segment was utilized to identify eyes with induced OHT after DEX treatment in one eye of a paired eye, and the primary bovine TM cell cultures were established in the contralateral paired eye with known GC responsiveness. The observed GC response rate for the bovine eyes in perfusion organ culture was found to be 36.8% [13]. However, the findings from bovine eyes may not be extrapolated to human GC responsiveness due to anatomical and physiological variations [17,18,19]. Therefore, in the present study, the combination of both a human organ-cultured anterior segment (HOCAS) ex vivo model and in vitro model of primary human TM (HTM) cells with known GC responsiveness were adopted to investigate differential gene expression using genome-wide transcriptome analysis with RNA sequencing (RNA-seq).

Primary HTM cells with known GC responsiveness were assessed using the human organ-cultured anterior segment (HOCAS) ex vivo model system to identify eyes with induced GC-OHT [16]. In a paired eye, one eye was used to determine the GC responsiveness following dexamethasone (DEX) treatment in HOCAS and the other eye was used to establish cultured primary HTM cells. This is the first study reporting distinct gene signatures with their associated pathways for GC-responder (GC-R) and non-responder (GC-NR) HTM cells. The data from this study has identified genes and pathways which are potential therapeutic targets to treat the underlying molecular pathology and mechanisms of GC-induced ocular hypertension (GC-OHT) and glaucoma (GIG).

## 2. Materials and Methods

### 2.1. Human Donor Eyes

Post-mortem human cadaveric eyes unsuitable for corneal transplantation were obtained from the Rotary Aravind International Eye Bank, Aravind Eye Hospital, Madurai. The tissues were handled in accordance with the Declaration of Helsinki after obtaining approval from the standing Human Ethics Committee of the Institute. The donor eyes were enucleated within 5 h of death (mean elapsed time between death and enucleation was 2.75 ± 1.58 h) and kept at 4 °C in the moist chamber until culture. All eyes were examined under the dissecting microscope for any gross ocular pathological changes and only macroscopically normal eyes were used for the experiments. The information related to phakic/aphakic status, as well as the history and duration of diabetes for the donor eyes used in the present study was available, but the other data related to systemic illness were not available.

In a set of paired eyes, one eye was used to establish a HOCAS ex vivo model system to characterize GC responsiveness after DEX treatment and the other eye was used to establish primary HTM cultures from eyes with identified responsiveness [16] (Figure S1). The characteristics of donor eyes used for this study are summarized in Table S1a.

### 2.2. Human Organ-Cultured Anterior Segment (HOCAS)

In a set of paired eyes, one eye was used to establish a HOCAS, as described previously [16,20]. Briefly, after baseline equilibration (~72 h) one eye of each pair received 5 mL of 100 nM DEX for 7 days. The eye pressure was monitored continuously using pressure transducers (APT 300 Pressure Transducers, Harvard Apparatus, Holliston, MA, USA) with data recorder (Power Lab system (AD Instruments, NSW, Bella Vista, Australia) and LabChart Pro software (ver.8.1). The intraocular pressure (IOP) was calculated every hour as the average of 6 values recorded every 10 min, beginning 4 h before the drug infusion and continuing for the duration of the culture. The average IOP in the 4 h before drug infusion was taken as the baseline IOP for calculation. Mean IOP was calculated for every day after respective treatments. Then, ΔIOP was calculated using the formula: actual IOP averaged over 24 h—basal IOP of individual eyes on certain day [16,21]. The increase in IOP in response to DEX treatment was examined for all treated eyes. The eyes were categorized as GC-responder (GC-R: mean ΔIOP was >5 mmHg from the baseline) and non-responder eyes (GC-NR: mean ΔIOP < 5 mmHg from the baseline) after DEX treatment for 7 days, as described earlier [22].

### 2.3. Primary Human TM Cell Strain with Known GC Responsiveness

The TM tissue was excised from the other eye of each set of paired eyes and the cell culture was established by the extracellular matrix digestion method, as described previously [23,24]. Primary HTM cells were grown at 37 °C in 5% CO_2_ in low glucose Dulbecco’s Modified Eagle Medium (DMEM) with 15% fetal bovine serum, 5 ng/mL basic fibroblast growth factor and antibiotics. The primary HTM cells isolated from the other eye of each pair was characterized with aquaporin, myocilin and phalloidin staining by immunofluorescence analysis (Figure S2). HTM cell strains with more than 50% myocilin positivity were used for further experiments [25]. Confluent cultures of GC-R and GC-NR HTM cells were serum-starved for 24 h and then treated with either 100 nM DEX or 0.1% ethanol (ETH) as a vehicle control for 7 days in media lacking both FGF and FBS. HTM cells from passages 2–4 were used for all experiments. Following DEX and 0.1% ETH treatment for 7 days, cultured HTM cells isolated from GC-R (*n* = 4) and GC-NR (*n* = 4) paired eyes from the HOCAS model were subjected to RNA extraction and RNA sequencing (Table S1b).

### 2.4. RNA Extraction and mRNA Sequencing

Total RNA was isolated from treated primary HTM cells by using the TRIZOL reagent (Sigma, St. Louis, MO, USA) as per the manufacturer’s instructions. RNA quantity and quality were assessed by NanoDrop 1000 spectrophotometer (Thermofisher Scientific, Delaware, USA), and TapeStation (Agilent Technologies, Santa Clara, CA, USA), respectively. Additionally, the quality of RNA was observed by the ratio of 28S and 18S ribosomal bands on 0.8% agarose gel electrophoresis. The samples with an RNA Integrity Number (RIN) value greater than 7 were used for RNA sequencing.

RNA sequencing for transcriptome profiling was performed at the Sandoor Lifesciences, Hyderabad, India. Briefly, 1 μg of total RNA was used to enrich mRNA using NEB Magnetic mRNA isolation kit according to the manufacturer’s instructions (NEB, Ipswich, MA, USA). cDNA was synthesized and ligated to sequence adapters. The transcriptome library was prepared using the NEB Ultra II RNA library preparation kit as per the manufacturer’s recommended protocol (NEB, Ipswich, MA, USA) and the libraries then underwent size selection, PCR amplification, and then PAGE purification. The final enriched libraries were purified and quantified by Qubit (Thermofisher Scientific, Burlington, UK) and size analyzed by Bio-analyzer (Agilent Technologies, Santa Clara, CA, USA). The resulting libraries were indexed and pooled then sequenced using Illumina Next Seq 500 (150 bp paired-end sequencing). Approximately 20–35 million reads were generated from each sample and obtained by demultiplexing.

### 2.5. Mapping and Differential Expression Analysis

The quality of raw reads was assessed by FastQC toolkit (http://www.bioinformatics.babraham.ac.uk/projects/fastqc/) (accessed on 18 November 2019), and adapter sequences were removed using bbduk.sh shell script from bbmap short read aligner (https://jgi.doe.gov/data-and-tools/bbtools/bb-tools-user-guide/bbmap-guide/) (accessed on 30 December 2019). The pre-processed high-quality reads were then mapped with human reference genome assembly GRCh38/hg38 using HISAT2 by following the default parameters [26]. The mRNA abundance in read counts were estimated using FeatureCounts [27]. MRNAs with less than 10 read counts were excluded from further analysis. The read counts were then normalized using a quantile strategy and the differential expression analysis with fragments per kilobase of exon per million (FPKM) values was performed by an R package: edgeR [28]. The mRNAs were considered as differentially expressed if the absolute fold change (log2) value was >2 and the *p* value < 0.05. Bonferroni correction was used to calculate the adjusted *p* value within the EdgeR package. For comparison, the differentially expressed genes (DEGs) were segregated into five groups, as previously described [16]: Group #1: DEGs between DEX and ETH treated GC-R HTM cells; Group #2 DEGs between DEX and ETH treated GC-NR HTM cells; Group #3: DEGs that are common to Group #1 and Group #2; Group #4: Uniquely expressed DEGs of GC-R HTM cells (Group #1 minus Group #3); Group #5: Uniquely expressed DEGs of GC-NR HTM cells (Group #2 minus Group #3). 

### 2.6. Pathway Enrichment Analysis 

Pathways associated with DEGs were enriched using the Database for Annotation, Visualization, and Integrated Discovery (DAVID) [29] with the KEGG database. The pathways with a fold enrichment of above 1 or below −1 with *p* values less than 0.05 were considered as significantly altered. The altered pathways were then clustered into their functional categories based on molecular mechanisms. 

### 2.7. Validation of RNA Seq by RT^2^-Profiler PCR Array

The expressions of the most up-regulated and down-regulated genes from Group #3, Group #4 and Group #5 identified in the RNA-Seq were further validated by a RT^2^-Profiler PCR array (Qiagen, Hilden, Germany), as per the manufacturer’s instructions. A list of genes taken for expression validation by the RT^2^-Profiler PCR array is shown in Table S2. Briefly, the total RNA from the TM cells after DEX treatment was isolated using RNAeasy mini kit (Qiagen, Hilden, Germany) and reverse-transcribed into cDNA using RT2 first-strand cDNA synthesis kit (Qiagen, Hilden, Germany), according to the manufacturer’s instructions. The PCR array was performed in a total volume of 25 µL containing 25 ng of total cDNA and 5× SYBR green master mix loaded in each well containing gene-specific probes along with the reference controls: ACTB, B2M and GAPDH genes. The PCR array was performed by three steps of a cycling program: 95 °C for 10 min for 1 cycle, followed by 40 cycles of 95 °C for 15 s, and 60 °C for 60 s, using the ABI-QuantStudio 5 (Applied Biosystems, Waltham, MA, USA). The expression of genes in DEX treated HTM cells in logFC ratio was calculated by normalizing with the reference control and vehicle control.

### 2.8. Statistical Analysis

Statistical analysis was carried out using Graph Pad Prism (ver.8.0.2) (Graph Pad software, San Diego, CA, USA). All data are presented as mean ± SEM or otherwise specified. Statistical significance between two groups (treated vs. vehicle control (ETH)) was analyzed using the paired or unpaired 2-tailed Student’s t-test. *p* < 0.05 or less was considered as statistically significant.

## 3. Results

### 3.1. Establishment of GC-R and GC-NR HTM Cells

In this study, one eye of each set of paired eyes was used to assess the GC responsiveness in HOCAS after 100 nM DEX treatment and the other eye was used to establish the primary HTM cells. HTM cells were characterized with known markers such as *MYOC* expression and CLAN formation after DEX treatment for 7 days [25] (Figure S2).

**Figure 1:**
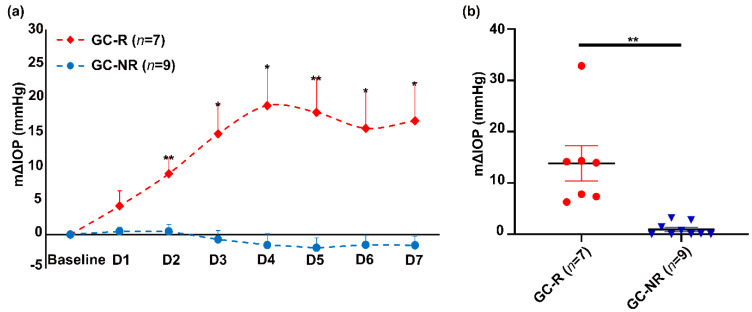
**Effect of DEX on IOP** (**a**) The mean ± SEM of ∆IOP of DEX-treated responder and non-responder eyes were plotted over time. The basal IOP on day 0 (before DEX treatment) was set at 0 mmHg. Treatment with DEX showed a significant elevated IOP in 7/16 eyes (Mean ± SEM-mΔIOP: 13.8 ± 3.41 mmHg; response rate: 43.8%). (**b**) Frequency plot of IOP data. The m∆IOP of GC-R and GC-NR groups were plotted. The m∆IOP was increased after DEX treatment in GC-R eyes as compared to GC-NR eyes. Data were analyzed by unpaired 2-tailed Student’s t-test on each treatment day; * *p* < 0.05; ** *p* < 0.001.

Based on the IOP response, the HTM cells established from each donor eye were categorized as GC-R and GC-NR cells. In HOCAS, DEX treatment caused an elevated IOP in 7/16 eyes (43.8%) (Mean Δ (±SEM) IOP: GC-R eyes: 13.8 ± 3.4 mmHg and GC-NR eyes: 0.91 ± 0.4 mmHg) (Figure 1). The raw data of IOP of all the studied eyes are summarized in Table S3. 

### 3.2. RNA Seq Data Quality

The fastQC evaluation of RNA seq revealed that the Phred score of all reads (forward and reverse) met the expected criteria of >30 (99.9% base call accuracy). After adapter and PCR duplicate trimming, approximately 5–7% of reads were excluded from the further analysis. In addition to HTM cells with known GC responsiveness, primary HTM cells (*n* = 2) cultured in DMEM media for 7 days were included to assess the effect of ETH on the expression of the genes; no significant changes between media treated and ETH-treated cells were found.

### 3.3. Differentially Expressed Genes of GC-R and GC-NR HTM Cells 

**Figure 2:**
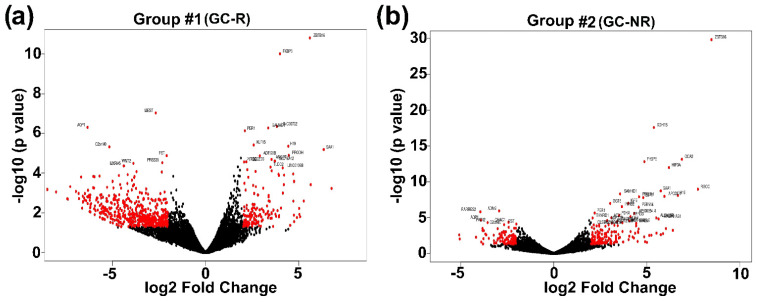
**Volcano plot showing the distribution of DEGs** The fold of change (log2) and *p*-value (-log10) of differentially expressed genes from (**a**) Group #1 and (**b**) Group #2 are represented. Red color indicates the significantly dysregulated genes with an absolute fold change >2 and *p*-value: <0.05. Note: Group #1: DEGs between DEX- and ETH-treated GC-R HTM cells; Group #2 DEGs between DEX- and ETH-treated GC-NR HTM cells.

An average of 85.6% of mRNA reads were aligned with the human reference genomes from the HTM cells used in the present study. The details of RNA sequencing and alignment statistics are shown in Table S4. The total number of genes identified in HTM cells of each donor eye ranged from 14,515 to 17,371. Principal component analysis of normalized data demonstrated that the DEX-treated cells were dispersed from the ETH-treated cells (Figure S3). The expression of DEGs from GC-R (Group #1) and GC-NR (Group #2) HTM cells are represented in volcano plot (Figure 2). 

**Figure 3:**
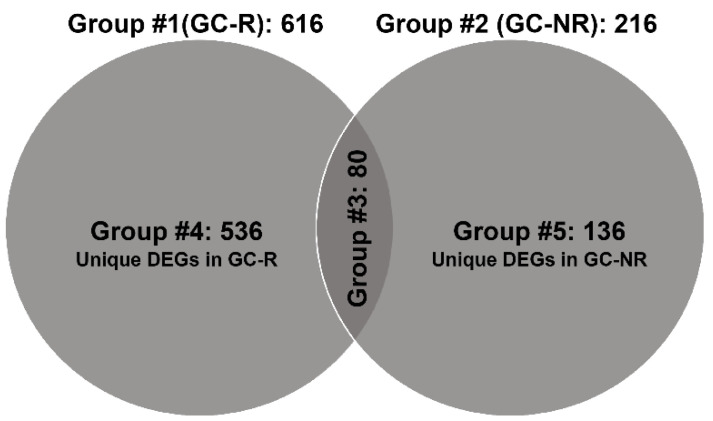
Venn diagram showing differentially expressed gene (DEG) groupings. DEGs of three groups from RNA seq data are shown; only genes with absolute fold change >2 and significant *p* value < 0.05 were included in these groupings. Note: Group #1: DEGs between ETH- and DEX-treated cells of GC-R HTM cells; Group #2: DEGs between ETH- and DEX-treated cells of GC-NR HTM cells; Group #3: common genes between Group #1 and Group #2; Group #4: uniquely expressed genes in GC-R; and Group #5: uniquely expressed genes in GC-NR.

In total, there were 616 and 216 DEGs in Group #1 (106 up-regulated; 510 down-regulated) and Group #2 (129 up-regulated; 87 down-regulated), respectively. There were 80 common DE genes found in Group #3 with an absolute fold change (log2) value > 2, and the *p* value < 0.05. In total, 536 (56 up-regulated; 480 down-regulated) and 136 (78 up-regulated; 58 down-regulated) DE genes were found to be uniquely expressed only in GC-R (Group #4) and GC-NR (Group #5) HTM cells, respectively (Figure 3). In Group #4, *SAA4* (log FC = 4.75), *FRG2C* (log FC = 5.27) and *NTRK2* (log FC = 3.39) were significantly up-regulated, and *UPK3A* (log FC = −8.48), *RLN1* (logFC = −8.01) and *NPY* (logFC = −7.2) were significantly down-regulated. In Group #5, *FAM107A* (logFC = 4.43), *STEAP4* (logFC = 4.57), *RGCC* (logFC = 7.75) were significantly up-regulated, and *GRM5* (logFC= −5.05), *SLC24A2* (logFC = −3.89) and *GRIA2* (logFC = −3.82) were significantly down-regulated. In Group #3, the common DEGs in Group #1 and #2 were *SAA1*, *ZBTB16*, *FKBP5* and *MYOC* (up-regulated), and *AQP1* and *LAMP3* (down-regulated). 

The top 50 DEGs from Group #1–Group #5 are shown in Table S5a–e, respectively. The top 10 DEGs from Group #3–#5 are given in Table 1.

In the present study, it is interesting to note that a number of genes involved in WNT signaling were down-regulated in TM cells after DEX treatment as compared to vehicle control and upon comparing between TM cells isolated from the GC-R and GC-NR eyes. Specifically, the WNT signaling genes (*WNT2* (logFC = −3.86), *WNT4* (logFC = −2.48), *WNT6* (logFC = −2.27), *WNT7B* (logFC = −3.84), *WNT10A* (logFC = −4.03), *WNT10B* (logFC = −3.03), *WNT11* (logFC = −2.18)) and WNT signaling antagonist secreted frizzled-related proteins (sFRP2 (logFC = −4.09) and sFRP4 (logFC = −2.13)) was down-regulated in GC-R HTM cells (Group #4). In Group #5, *WNT2* (logFC = −2.69) was identified as the only WNT signaling gene with a down-regulated expression in GC-NR HTM cells. 

### 3.4. Pathway Analysis

The pathway analysis of DEGs from Group #3, Group #4 and Group #5 identified 75, 64 and 46 altered pathways, respectively. The altered pathways of each group were clustered into multiple functional categories. In Group #3, focal adhesion, WNT signaling, MAPK signaling, TGFβ signaling, drug metabolism cytochrome, cell adhesion, and pathways in cancer were found as most commonly enriched between GC-R and GC-NR HTM cells. The adherens junction, T cell receptor signaling, B cell receptor signaling, the chemokine signaling pathway, the regulation of actin cytoskeleton, and the TNF signaling pathways were enriched uniquely in GC-R HTM cells (Group #4). Interestingly, the predominant down-regulation of axon guidance, ECM-receptor interaction, focal adhesion, the PI3K-Akt signaling pathways, and the up-regulation of the calcium signaling pathway and vascular smooth muscle contraction were identified in Group #5, compared to Group #4. The results of pathway analysis for the studied groups are summarized in Table S6a–e.

### 3.5. Validation of DE genes by RT^2^-PCR Array

**Figure 4:**
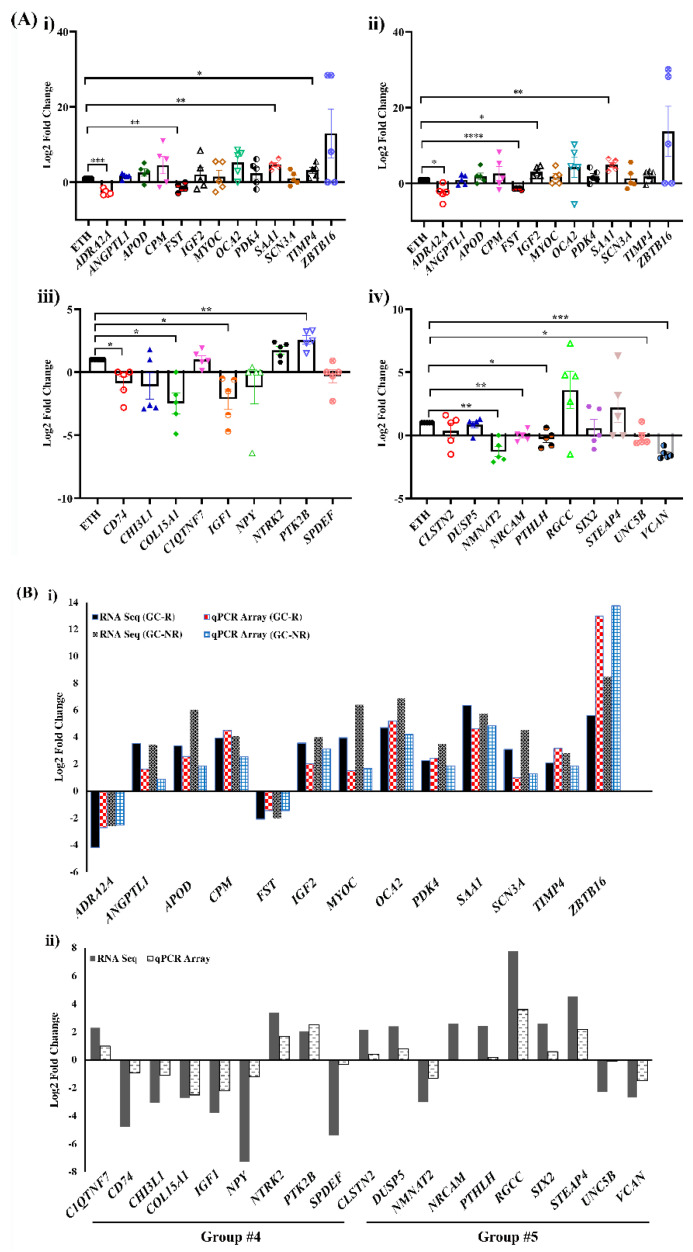
(**A**) **Validation of DEGs by RT^2^-PCR Array.** Expression profile of selected genes identified from RNA-seq and validated by RT^2^-Profiler PCR array is shown. Primary HTM cells were treated with 100 nM DEX or 0.1% ETH for 7 days. Total RNA was extracted, converted to cDNA, and the expression profile of selected genes were carried out by RT^2^ -PCR array as per the manu-acturer’s instructions (refer to the methods). Gene expressions were normalized to *ACTB* and ana-lyzed using the 2^−ΔΔCT^ method followed by Log_2_FC calculation. (i) Expression profile of selected genes from Group #3-GC-R, (ii) Group #3-GC-NR, (iii) Group #4, and (iv) Group #5 are shown. The data are represented as mean ± SEM. * *p* < 0.05; ** *p* < 0.01; *** *p* < 0.001, **** *p* < 0.0001. Paired 2-tailed Student’s t test. (**B**) **Validation of RNA sequencing findings by RT^2^-PCR Array.** Out of 32 genes, the expression pattern identified from RNA-seq of 30 genes matched with RT^2^ -PCR array analysis. DEGs from (i) Group #3, (ii) Group #4, and Group #5.

Out of 32 genes selected for PCR array, the expression pattern of 30 genes matched with RNA seq data, which further confirmed the reliability of these two techniques (Figure 4A,B). As *GAPDH* and *B2M* showed significant changes to steroid treatment in at least one biological sample from each group, *ACTB* was used as a reference control.

## 4. Discussion

The pathophysiological mechanisms causing OHT and glaucoma associated with the use of GCs are not clearly understood. However, the structural and functional alterations in the TM associated with GC have been well documented [30]. Gene expression studies after DEX treatment in cultured HTM cells [7,8,9,10,11,12,13,14] and perfusion-cultured bovine anterior segments [16] have identified differentially expressed genes associated with GC exposure in the TM (Table S7). It is important to dissect generic alterations in gene expression associated with GC exposure in the TM from the genes implicitly involved in GC-induced OHT and GIG: i.e., GC responsiveness. Previous studies have identified only global changes in gene expression in response to DEX treatment, but not on the specific genes and pathways implicated in GC responsiveness and raised IOP in human eyes [7,8,9,10,11,12,13,14]. Neither the IOP response nor the presence of GC responsiveness of the HTM cells/tissues derived from the donor eyes were known in previously reported studies; hence, there were inconsistencies in differential gene expression. Therefore, in the present study, the differentially expressed genes in primary HTM cells with known GC responsiveness were investigated using RNA-seq technology. 

Uniquely, in this study, the HOCAS ex vivo model was used to identify eyes with induced GC-OHT after DEX treatment based on the maximum IOP change (>5 mmHg): GC responsiveness. Eyes could then be classified as GC-R or GC-NR based on a pathophysiological IOP response in the HOCAS model with DEX treatment. Primary HTM cell cultures were established from the contralateral paired donor eyes after the identification of GC responsiveness in HOCAS, which provided cultured HTM cells with known GC responsiveness and represented a unique resource to explore the molecular basis of GC-OHT and GIG. A cell culture model was established as the yield of total RNA from individual TM tissue after the HOCAS experiment was limited for the RNA-seq experiment. Therefore, cultured HTM cells treated with DEX for 7 days from the paired eye with known GC-R and GC-NR status were used for RNA-seq. In order to validate the characteristics of primary HTM cells used in the present study, the expression of MYOC and CLAN formation after DEX induction were used. The cells with more than 50% MYOC positivity were used for all of our analyses [25]. To our knowledge, this is the first study reporting the differentially expressed genes in primary HTM cells with known GC responsiveness using RNA-seq technology. 

The results of the present study revealed that an average of 16,022 genes were identified in cultured HTM cells, out of which the significantly altered genes in Group #3, #4 and #5 were 80, 536 and 136, respectively. A higher number of significantly altered genes were found in GC-R cells (Group #1) (616 genes) as compared to GC-NR HTM cells (Group #2) (216 genes) in response to DEX treatment, which indicates that both cells behaved differently to GC treatment. 

A group of common genes which had been reported in previous studies to be up-regulated by DEX treatment in HTM cells were also found in our study [7,8,9,10,11,12,13,14] (Table S7). The DEGs found in Group #4 were specifically relevant to GC-OHT/GIG, as these genes were uniquely expressed DEGs of GC-R HTM cells. The apolipoprotein E (*APOE*) is the major lipoprotein in the central nervous system which plays an important role in the uptake and distribution of cholesterol within the neuronal network. The polymorphism of *APOE* has been reported previously with an increased risk of POAG [31,32]. Our study showed that APOE was up-regulated only in GC-R cells (logFC= 1.86) and not in the GC-NR group. Neuropeptide Y (*NPY*) is involved in various physiological and homeostatic processes in both the central and peripheral nervous systems. It is expressed in the retina of both mammalian and non-mammalian species [33]. The immunohistochemical staining of *NPY* has also been detected in the drainage angle of the mammalian eye [34]. *NPY* is reported to prevent neuronal cell death in the retina induced by excitotoxic insults. Interestingly, in the present study, it was highly down-regulated (logFC = −7.2) only in GC-R TM cells. Further studies are warranted to understand the role of *NPY* in determining differential GC responsiveness in HTM cells. 

The enrichment analysis with DEGs identified the pathways previously reported in glucocorticoid-treated TM cells, as well as additional pathways enriched in the present study after DEX treatment (Table S8a). Of 11 functional pathways that were reported to be significantly altered in human TM cells after exposure to DEX treatment versus a control medium, only 2 pathways (cell adhesion and WNT signaling pathways) were replicated in the present study [35]. The other previously reported pathways did not show any significant change in the present study [35]. Uniquely, in our study, we were able to sub-analyze the pathways enriched between the GC-R and GC-NR HTM cells (Table S8b). One previous study had reported enriched pathways in bovine GC-R and GC-NR TM cells [16]. Cell cycle and senescence pathways were highly significant between bovine GC-R and GC-NR TM cells. These pathways did not differ between human GC-R and GC-NR TM cells but, interestingly, our pathway analysis identified focal adhesion, WNT signaling, MAPK signaling, TGFβ signaling, drug metabolism cytochrome P450 and cell adhesion pathways to be associated with GC responsiveness (Table S6b). This variation may be attributed to the species variation and their corresponding responsiveness to DEX treatment. 

In the current study, it is interesting to note that the WNT signaling pathway was uniquely down-regulated in the TM cells of GC-R HTM cells. In general, dysregulated WNT signaling has been associated with glaucoma and the expression of WNT signaling antagonist sFRP1 is up-regulated in glaucomatous TM cells [36]. The up-regulation of sFRP1 was found to induce an elevated IOP in both organ culture and murine models [36]. Moreover, the WNT signaling pathway was found to mediate ECM expression [37] and TM cell stiffening [38] in cultured HTM cells. A WNT signaling small molecule inhibitor was effective in restoring the GC-induced phenotypic changes in TM cells, supporting a potential therapeutic application in steroid-induced glaucoma [39].

Several studies have shown inhibitory crosstalk between GCs and transforming growth factor β (TGF-β) [40,41], and the inhibition of TGF-β signaling by GCs is known to be mediated by either reducing the bio-availability of TGF-β or by regulating SMAD signaling [40,41,42]. Interactions between DEX and TGF-β signaling mediate GC-OHT as DEX activates TGF-β signaling, inducing ER stress and ECM alterations resulting in IOP elevation [43]. The present study also identified the up-regulation of TGF-β signaling in both GC-R (logFC = 1.65) and GC-NR (logFC = 1.38) human TM cells after DEX exposure which further confirms the observation of the previous study [43]. However, further investigations are warranted to decipher the functional role of TGF-β signaling in differential GC responsiveness. 

The major strengths of this study include the use of TM cells derived from human cadaveric eyes with known GC responsiveness identified in the HOCAS model to investigate transcriptome alterations in human TM cells responsible for GC responsiveness. The observed GC response rate in perfusion-cultured human cadaveric eyes of the present study was similar to a previous study reported by other groups [1,22] and our group [20]. As age is known to be a risk factor for GC-OHT/glaucoma, the donor eyes from young age groups were excluded from the study. RNA-seq technology enabled the identification of significantly altered genes and pathways in GC-R and GC-NR human TM cells for the first time. 

However, there are some limitations to the current study which need to be addressed. RNA directly extracted from the TM of the DEX-perfused human cadaveric eyes with known IOP response to GC treatment would be the ideal experimental design to investigate the transcriptional basis of GC responsiveness. Given the RNA quantity derived from TM tissues from donor eyes in our preliminary study was not sufficient to run a robust RNA-seq analysis, cultured HTM cells derived from the contralateral paired eyes of known GC responsiveness were utilized in the present study. The usage of cultured cells might have contributed to variations in gene expression, as compared to native tissues. In order to avoid such variability, HTM cells were cultured in growth media for 7 days and their gene expression profiles were compared with HTM cells grown in media containing 0.1% ethanol (vehicle control); no significant changes were found in the gene expression patterns. The history of glaucoma or any other ocular diseases of the human donor eyes used in the present study was not known, which could alter the expression profile of significantly altered genes between GC-R and GC-NR HTM cells. In addition, due to the limited availability of human donor eyes, some of the donor eyes used in the present study had postmortem times over 48 h; however, the viability of the tissues were maintained by storing them at 4 °C immediately after enucleation until culture. 

In conclusion, this is the first study reporting the differentially expressed genes in HTM cells with known GC responsiveness using RNA-seq technology. Utilizing perfusion-cultured human cadaveric eyes in an ex vivo model enabled the identification of the induction of GC-OHT after DEX treatment, and thus the classification of HTM cells based on GC responsiveness: GC-R and GC-NR. Some previously reported and unique genes and their associated pathways were identified in TM cells in response to DEX treatment; more significantly, the transcriptional changes unique to GC-R and GC-NR HTM cells were identified. This study supports the further study of the genes and proteins which were uniquely expressed by the GC-responder eyes in the Indian population. A further understanding of the molecular basis of HTM GC responsiveness could identify novel therapeutic approaches for GC-OHT and GIG, which is of direct clinical relevance given the widespread use of glucocorticoids in ophthalmology and medicine.

## Figures and Tables

**Table 1:** List of top 10 up/down-regulated genes.

**List of Genes from Group #3**								
**Gene**	**Group #1**			**Group #2**				
	**logFC**	**logCPM**	***p* Value**	**logFC**		**logCPM**		***p* Value**
*ZBTB16*	5.60	4.66	0.000	8.48		4.59		0.000
*OCA2*	4.71	1.61	0.000	6.89		2.24		0.000
*H19*	4.44	7.60	0.000	6.67		8.47		0.000
*MYOC*	3.95	6.57	0.014	6.40		9.28		0.001
*HIF3A*	4.20	4.11	0.000	6.19		2.63		0.000
*APOD*	3.35	7.28	0.018	6.03		8.49		0.000
*SAA1*	6.34	2.81	0.000	5.75		1.16		0.000
*ADH1B*	3.39	7.72	0.001	5.38		6.06		0.000
*FKBP5*	3.99	7.23	0.000	4.87		7.62		0.000
*LSP1*	3.91	5.22	0.000	4.81		4.97		0.000
**List of Genes from Group #4**								
**Up-regulated genes**				**Down-regulated genes**				
**Gene**	**logFC**	**logCPM**	***p* Value**	**Gene**	**logFC**		**logCPM**	***p* Value**
*FRG2C*	5.27	−2.07	0.002	*UPK3A*	−8.49		0.36	0.001
*NPSR1-AS1*	5.07	−2.19	0.016	*RLN1*	−8.02		1.24	0.001
*BHLHE22*	5.04	0.77	0.012	*FAM110D*	−7.42		−0.59	0.002
*SAA4*	4.75	−2.34	0.028	*PRSS22*	−7.38		−0.62	0.002
*TUSC5*	3.79	−1.37	0.015	*NPY*	−7.26		3.41	0.000
*LEP*	3.75	−0.16	0.012	*GIMAP1*	−6.96		−0.98	0.001
*RNA5SP111*	3.63	−2.00	0.049	*ST14*	−6.81		1.52	0.001
*KCNE1*	3.55	0.74	0.010	*LRRC26*	−6.79		1.53	0.002
*RPL7P57*	3.49	−2.06	0.019	*KRT15*	−6.67		3.39	0.000
*NTRK2*	3.39	5.15	0.002	*FXYD3*	−6.59		2.02	0.001
**List of Genes from Group #5**								
**Up-regulated genes**				**Down-regulated genes**				
**Gene**	**logFC**	**logCPM**	***p* Value**	**Gene**	**logFC**		**logCPM**	***p* Value**
*RGCC*	7.75	1.95	0.000	*GRM5*	−5.05		−2.43	0.010
*APCDD1*	5.95	2.15	0.000	*SLC24A2*	−3.89		−1.78	0.025
*IGF2-AS*	5.88	−1.83	0.002	*GRIA2*	−3.82		0.29	0.001
*PNMT*	5.76	−1.91	0.002	*RBFOX1*	−3.80		−1.82	0.048
*RAMP2-AS1*	5.64	0.83	0.000	*KRT17P1*	−3.77		−1.24	0.009
*ALOX15B*	5.51	−0.80	0.000	*GRID2*	−3.67		−0.01	0.002
*PTGDR2*	5.24	−2.22	0.003	*VCAN-AS1*	−3.63		−1.95	0.003
*LINC00525*	5.13	−2.29	0.003	*PADI2*	−3.55		0.96	0.000
*STEAP4*	4.57	3.40	0.000	*KLHDC7B*	−3.22		−0.85	0.003
*SFTPC*	4.51	−2.59	0.021	*IGFL2*	−3.20		−2.15	0.040

Note: *Group #3:* DEGs that are common to Group #1 and Group #2; *Group #4:* Uniquely expressed DEGs of GC-R HTM cells (Group #1 minus Group #3); *Group #5:* Uniquely expressed DEGs of GC-NR HTM cells (Group #2 minus Group #3).

## Data Availability

Data Access: The raw mRNA sequencing data of HTM cells from each human donor eye used in the present study have been deposited publicly in NCBI-SRA under the BioProject PRJNA729873. Code Availability: The bioinformatics In-house pipeline used for mRNA sequencing data analysis in the present study have been submitted to GitHub in shell script (https://github.com/SenthilKumariLab/mRNA-seq-Analysis-Pipeline.git) (accessed on 14 May 2021).

## Supplementary Materials

The following supporting information can be downloaded at: https://www.mdpi.com/article/10.3390/genes13050882/s1, Figure S1: Work plan showing the establishment of primary HTM cell strains with known GC responsiveness; Figure S2: Isolation and characterization of primary HTM cells; Figure S3: principle component analysis (PCA) of RNA-seq data from normalized read counts in comparison with DEX to ETH (vehicle); Table S1: Characteristics of human donor eyes used for the present Study; Table S2: List of genes selected from RNA sequencing for validation by RT2- PCR array; Table S3: The raw data of the IOP of perfusion-cultured Indian cadaveric eyes; Table S4: Alignment statistics of RNA sequencing data; Table S5a: List of top 50 up/down-regulated genes from Group #1; Table S5b: List of top 50 up/down-regulated genes from Group #2; Table S5c: List of overlapping genes from Group #3; Table S5d: List of top 50 up/down-regulated genes from Group #4; Table S5e: List of top 50 up/down-regulated genes from Group #5; Table S6a: List of enriched pathways in Group #1; Table S6b: List of enriched pathways in Group #2; Table S6c: List of enriched pathways in Group #3; Table S6d: List of enriched pathways in Group #4; Table S6e: List of enriched pathways in Group #5; Table S7: Comparison of differentially expressed genes in TM cells treated with glucocorticoid from previous reports and the present study; Table S8a: Comparison of pathways involved in DEX-treated TM Cells from previous reports and the present study; Table S8b: Comparison of pathways enriched in responder and non-responder HTM cells from previous report and the present study.
