# Supplementary material for: A Comparative Genome-Wide Transcriptome Analysis of Glucocorticoid Responder and Non-Responder Primary Human Trabecular Meshwork Cells
Source: Genes (Basel). 2022 May 15;13(5):882. doi: 10.3390/genes13050882 (PMC9140469; doi:10.3390/genes13050882)
Supplement: Supplementary file 1 [file genes-13-00882-s001.zip › genes-1668080-supplementary/genes-1668080/Supplementary Table 27.4.22.pdf]

Supplementary Table S1a: Characteristics of Human Donor Eyes Used for the Present Study

DEX Treatment (nM)	Code	Age	Sex	Cause of Death	Time B/W Death & Enucleation (h)	Time B/W Enucleation & Culture (h)	Experiment Eye	HOCAS/ Culture	Treatment	Remarks
100nM (N=16)	OCHD18-29	76	F	Natural	0.45	30.5	OD	HOCAS	DEX	Data included
							OS	Cell culture	ETH/DEX	TM Culture established
	OCHD18-49	48	M	Heart Disease	2.5	30.66	OD	HOCAS	DEX	Data included
							OS	Cell culture	ETH/DEX	TM Culture established
	OCHD18-52	55	F	Vascular accident	2.75	67.91	OD	HOCAS	DEX	Data included
							OS	Cell culture	ETH/DEX	TM Culture established
	OCHD18-53	67	M	Diabetes	4.33	27	OD	HOCAS	DEX	Data included
							OS	Cell culture	ETH/DEX	TM Culture established
	OCHD18-56	82	M	Cardiac arrest	4	23	OD	HOCAS	DEX	Data included
							OS	Cell culture	ETH/DEX	TM Culture established
	OCHD18-57	80	F	Respiratory disease	3	28.5	OD	HOCAS	DEX	Data included
							OS	Cell culture	ETH/DEX	TM Culture established
	OCHD19-01	73	M	Natural	4.5	30.8	OD	HOCAS	DEX	Data included
							OS	Cell culture	ETH/DEX	TM Culture established
	OCHD19-02	66	M	Road traffic injury	2.75	8.33	OD	HOCAS	DEX	Data included
							OS	Cell culture	ETH/DEX	TM Culture established
	OCHD19-03	65	F	Heart Disease	5.8	47.33	OD	HOCAS	DEX	Data included
							OS	Cell culture	ETH/DEX	TM Culture established
	OCHD19-04	72	M	Heart Disease	3.5	41	OD	HOCAS	DEX	Data included
							OS	Cell culture	ETH/DEX	TM Culture established
	OCHD20-04	65	M	Chronic obstructive	0.33	28.5	OD	HOCAS	DEX	Data included
							OS	Cell culture	ETH/DEX	TM Culture established

			pulmonary disease						
OCHD20-05	50	M	Kidney failure	2	71.58	OD	HOCAS	DEX	Data included
						OS	Cell culture	ETH/DEX	TM Culture established
OCHD20-06	59	M	Chronic Kidney Disease	0.5	29.16	OD	HOCAS	DEX	Data included
						OS	Cell culture	ETH/DEX	TM Culture established
OCHD20-09	46	M	Accidental fall	2.25	31	OD	HOCAS	DEX	Data included
						OS	Cell culture	ETH/DEX	TM Culture established
OCHD20-10	38	M	Chronic Kidney Disease	1.41	47.5	OD	HOCAS	DEX	Data included
						OS	Cell culture	ETH/DEX	TM Culture established
OCHD20-14	56	M	Heart Disease	3.91	72.25	OD	HOCAS	DEX	Data included
						OS	Cell culture	ETH/DEX	TM Culture established
**HTM Cell used for RNA-Sequencing**									
OCHD18-39	82	F	Respiratory disease	0.75	-	OS	Cell culture	ETH/DEX	TM Culture established

A total of 16 paired eyes were used for the study with the mean (±) SD age was 62.38 ± 12.82 years. The mean (±) SD elapsed time between (b/w) death and enucleation was 2.75 ± 1.58 h and the mean elapsed time between enucleation and culture was 38.44 ± 18.39 h. DEX- Eyes treated with dexamethasone; ETH/DEX- HTM Cells treated with either 0.1% ethanol or dexamethasone. * HTM Cells used for RNA-sequencing.

Supplementary Table S1b: Trabecular Meshwork Cells Used for the RNA Sequence

R/NR	Code	Experiment Eye	Treatment	RAN Seq
R	OCHD18-39	OS	ETH (0.1%)	Yes
			DEX (100nM)	Yes
	OCHD18-53	OS	ETH (0.1%)	Yes
			DEX (100nM)	Yes
	OCHD19-03	OS	ETH (0.1%)	Yes
			DEX (100nM)	Yes
	OCHD19-04	OS	ETH (0.1%)	Yes
			DEX (100nM)	Yes
NR	OCHD18-29	OS	ETH (0.1%)	Yes
			DEX (100nM)	Yes
	OCHD18-52	OS	ETH (0.1%)	Yes
			DEX (100nM)	Yes
	OCHD18-56	OS	ETH (0.1%)	Yes
			DEX (100nM)	Yes
	OCHD19-02	OS	ETH (0.1%)	Yes
			DEX (100nM)	Yes

Note: Responder; NR- Non-responder

Supplementary Table S2. List of Genes Selected from RNA Sequencing for Validation by RT^2- PCR Array

S.No	Ref seq	Symbol	Description
1	NM_000681	*ADRA2A*	Adrenergic, alpha-2A-, receptor
2	NM_004673	*ANGPTL1*	Angiopoietin-like 1
3	NM_001647	*APOD*	Apolipoprotein D
4	NM_031911	*C1QTNF7*	C1q and tumor necrosis factor related protein 7
5	NM_004355	*CD74*	CD74 molecule, major histocompatibility complex, class II invariant chain
6	NM_001276	*CHI3L1*	Chitinase 3-like 1 (cartilage glycoprotein-39)
7	NM_022131	*CLSTN2*	Calsyntenin 2
8	NM_001855	*COL15A1*	Collagen, type XV, alpha 1
9	NM_001874	*CPM*	Carboxypeptidase M
10	NM_004419	*DUSP5*	Dual specificity phosphatase 5
11	NM_006350	*FST*	Follistatin
12	NM_000618	*IGF1*	Insulin-like growth factor 1 (somatomedin C)
13	NM_000612	*IGF2*	Insulin-like growth factor 2 (somatomedin A)
14	NM_000261	*MYOC*	Myocilin, trabecular meshwork inducible glucocorticoid response
15	NM_015039	*NMNAT2*	Nicotinamide nucleotide adenylyltransferase 2
16	NM_000905	*NPY*	Neuropeptide Y
17	NM_005010	*NRCAM*	Neuronal cell adhesion molecule
18	NM_006180	*NTRK2*	Neurotrophic tyrosine kinase, receptor, type 2
19	NM_000275	*OCA2*	Oculocutaneous albinism II
20	NM_002612	*PDK4*	Pyruvate dehydrogenase kinase, isozyme 4
21	NM_002820	*PTHLH*	Parathyroid hormone-like hormone
22	NM_004103	*PTK2B*	PTK2B protein tyrosine kinase 2 beta
23	NM_014059	*RGCC*	Regulator Of Cell Cycle
24	NM_000331	*SAA1*	Serum amyloid A1
25	NM_006922	*SCN3A*	Sodium channel, voltage-gated, type III, alpha subunit
26	NM_016932	*SIX2*	SIX homeobox 2
27	NM_012391	*SPDEF*	SAM pointed domain containing ets transcription factor
28	NM_024636	*STEAP4*	STEAP family member 4

29	NM_003256	*TIMP4*	TIMP metallopeptidase inhibitor 4
30	NM_170744	*UNC5B*	Unc-5 homolog B
31	NM_004385	*VCAN*	Versican
32	NM_006006	*ZBTB16*	Zinc finger and BTB domain containing 16

Supplementary Table S3: The Raw Data of the IOP of Perfusion Cultured Indian Cadaveric Eyes

Dose group (nM)	R/NR	ID	Stabilization period (h)	Pressure change before and after drug treatment (mmHg)								mΔ IOP (mmHg)
				D0	D1	D2	D3	D4	D5	D6	D7	
100	R	OCHD18-53	51.3	22.89	30.72	35.47	49.43	54.96	39.82	22.77	24.63	13.94
		OCHD18-57	40.8	17.57	16.47	17.94	18.62	18.16	22.73	35.99	37.21	6.30
		OCHD19-03	43.1	18.20	33.94	34.10	32.25	30.54	31.32	32.50	32.96	14.32
		OCHD19-04	35.5	21.62	21.71	23.49	28.47	33.03	33.80	33.61	31.81	7.80
		OCHD20-04	65.1	10.29	12.27	23.35	43.61	55.56	56.21	54.71	56.20	32.84
		OCHD20-09	37.8	11.94	15.47	24.34	20.79	22.40	23.70	12.74	15.63	7.36
		OCHD20-14	68.3	22.75	23.96	28.84	35.16	42.68	42.76	41.77	43.33	14.18
	Baseline IOP (IOP on day 0): mean= 17.89, SEM= 1.93, mΔ IOP: mean= 13.82, SEM= 3.4.10; N= 7											
	NR	OCHD18-29	62.3	22.61	23.48	24.43	23.22	22.20	23.13	23.05	23.82	0.72
		OCHD18-49	33.3	13.25	13.61	16.96	14.58	19.37	15.68	14.84	17.65	2.85
		OCHD18-52	48.1	20.75	20.86	23.22	24.63	25.66	25.12	25.32	22.98	3.22
		OCHD18-56	40.8	21.71	21.90	18.18	14.83	13.40	13.13	13.20	13.23	0 [a]
		OCHD19-01	55.6	11.73	16.28	12.19	15.07	10.34	11.59	14.53	11.65	1.36
		OCHD19-02	27	12.50	13.18	16.60	13.34	12.42	11.78	10.09	10.61	0.07
		OCHD20-05	50.5	19.27	18.91	20.26	19.39	17.28	16.14	15.92	16.08	0 [a]
		OCHD20-06	48.5	11.99	11.86	10.71	7.71	6.83	6.73	8.81	9.32	0 [a]
		OCHD20-10	47.8	15.45	13.42	10.89	10.01	7.97	8.44	9.94	9.65	0 [a]
	Baseline IOP (IOP on day 0): mean= 16.59, SEM= 1.7, mΔ IOP: mean= 0.91, SEM = 0.43; N= 9											

Note: [a] Minus values are given as 0; Day 0 represents the day before respective drug treatments; R - Responder; NR- Non-responder

Supplementary Table S4: Alignment Statistics of RNA Sequencing Data

S. No	R/NR	ID		mRNA			
				Total Reads (In millions)	% Of Mapped Reads	% Of Unmapped Reads	No. of Genes
1	R	18-39	ETH	21.1	85.08	14.92	15765
			DEX	29.3	84.79	15.11	17079
2		18-53	ETH	22.5	85.76	14.24	17371
			DEX	36.2	82.11	17.89	16415
3		19-03	ETH	23.4	84.91	15.09	15353
			DEX	30.5	87.00	13.00	16396
4		19-04	ETH	27.5	87.40	12.60	14515
			DEX	30.7	85.38	14.62	16334
5	NR	18-49	ETH	26.8	84.30	15.70	15439
			DEX	30.3	85.22	14.78	15637
6		18-52	ETH	23.1	84.42	15.58	14682
			DEX	28.4	86.27	13.73	15997
7		18-56	ETH	24.8	86.28	13.72	15852
			DEX	36.2	86.28	13.72	16654
8		19-02	ETH	23.4	88.60	11.40	16039
			DEX	32.7	85.84	14.16	16828
			**Mean**	**28**	**86**	**14.4**	**16022**

Supplementary Table S5a. List of top 50 Up/Down-regulated Genes from Group #1

Up-regulated genes				Down-regulated genes			
**Gene**	**logFC**	**logCPM**	***P* Value**	**Gene**	**logFC**	**logCPM**	***P* Value**
SAA1	6.34	2.81	0.000	*UPK3A*	-8.49	0.36	0.001
ZBTB16	5.60	4.66	0.000	*RLN1*	-8.02	1.24	0.001
FRG2C	5.27	-2.07	0.002	*FAM110D*	-7.42	-0.59	0.002
NPSR1-AS1	5.07	-2.19	0.016	*PRSS22*	-7.38	-0.62	0.002
BHLHE22	5.04	0.77	0.012	*NPY*	-7.26	3.41	0.000
SAA4	4.75	-2.34	0.028	*GIMAP1*	-6.96	-0.98	0.001
SAA2	4.71	0.71	0.003	*ST14*	-6.81	1.52	0.001
OCA2	4.71	1.61	0.000	*LRRC26*	-6.79	1.53	0.002
PRODH	4.46	3.17	0.000	*KRT15*	-6.67	3.39	0.000
H19	4.44	7.60	0.000	*FXYD3*	-6.59	2.02	0.001
PRR33	4.22	1.04	0.000	*PRAC1*	-6.58	-0.09	0.003
HIF3A	4.20	4.11	0.000	*HOXB13*	-6.57	2.99	0.003
P2RY14	4.14	1.13	0.001	*TPSB2*	-6.50	0.98	0.002
LINC01088	4.14	1.46	0.000	*ACPP*	-6.48	6.82	0.002
FKBP5	3.99	7.23	0.000	*LCN2*	-6.47	0.44	0.001
MYOC	3.95	6.57	0.014	*KLK3*	-6.39	7.58	0.002
CPM	3.93	4.02	0.000	*KCNH2*	-6.34	0.38	0.002
ANGPTL7	3.93	8.01	0.016	*AQP1*	-6.33	7.61	0.000
LSP1	3.91	5.22	0.000	*DOC2B*	-6.30	-0.33	0.001
LINC00702	3.82	3.76	0.000	*LINC01297*	-6.30	1.17	0.002
TUSC5	3.79	-1.37	0.015	*TPSAB1*	-6.26	0.71	0.001
LEP	3.75	-0.16	0.012	*RBBP8NL*	-6.22	-1.54	0.009
SLC16A12	3.72	1.63	0.000	*CHRNA2*	-6.21	1.31	0.002
RNA5SP111	3.63	-2.00	0.049	*SLC52A3*	-6.20	-0.35	0.002
IGF2	3.56	6.66	0.000	*KLK4*	-6.20	3.10	0.006
KCNE1	3.55	0.74	0.010	*KRT5*	-6.19	2.48	0.001
ANGPTL1	3.54	3.59	0.000	*MT1H*	-6.18	-1.57	0.005
RPL7P57	3.49	-2.06	0.019	*KLK2*	-6.17	6.41	0.004
TLDC2	3.48	1.28	0.000	*CCDC64B*	-6.03	0.46	0.001
NTRK2	3.39	5.15	0.002	*SFN*	-6.02	1.83	0.000
ADH1B	3.39	7.72	0.001	*DES*	-5.98	6.03	0.001
FMO2	3.38	3.38	0.002	*MSMB*	-5.97	4.06	0.006
SAMHD1	3.36	7.81	0.000	*COL9A1*	-5.96	2.75	0.000
GIP	3.36	-1.29	0.008	*C2orf54*	-5.94	-1.73	0.012
APOD	3.35	7.28	0.018	*PAGE4*	-5.94	0.47	0.004
RN7SKP69	3.30	-1.68	0.004	*EVX2*	-5.90	-1.76	0.008
MIR5690	3.22	-1.75	0.012	*PLVAP*	-5.87	1.64	0.002
SCN3A	3.09	0.86	0.008	*RAMP3*	-5.83	0.30	0.002
TNFAIP8L3	3.09	3.20	0.001	*RAB25*	-5.81	0.28	0.002
MAP1LC3C	3.01	3.54	0.026	*HOXA9*	-5.78	-0.78	0.007
FHL5	2.99	-0.36	0.016	*CHRM1*	-5.77	0.88	0.003

SEMG1	2.97	-1.56	0.047	*PDE9A*	-5.68	1.33	0.000
ABRA	2.93	0.31	0.012	*SHH*	-5.67	-1.91	0.010
ADRA1B	2.91	2.49	0.000	*GIMAP7*	-5.67	-0.28	0.004
AFF2	2.90	1.40	0.007	*SPOCK3*	-5.64	1.74	0.001
ADH1A	2.85	1.95	0.014	*LCN6*	-5.61	-1.94	0.011
SLC16A10	2.84	1.28	0.028	*GNA15*	-5.60	-0.92	0.002
XRCC6P2	2.82	-0.36	0.001	*HOXA10*	-5.59	0.11	0.001
BCRP1	2.82	-1.97	0.032	*MMP7*	-5.58	1.84	0.004
HSPD1P11	2.81	1.79	0.001	*GATA5*	-5.58	-0.87	0.009

Note: *Group #1*: DEGs between DEX and ETH treated GC-R HTM cells

Supplementary Table S5b. List of top 50 Up/Down-regulated Genes from Group #2

Up-regulated genes				Down-regulated genes			
**Gene**	**logFC**	**logCPM**	***P* Value**	**Gene**	**logFC**	**logCPM**	***P* Value**
ZBTB16	8.48	4.59	0.000	*MXRA5Y*	-5.08	-0.47	0.003
RGCC	7.75	1.95	0.000	*GRM5*	-5.05	-2.43	0.010
OCA2	6.89	2.24	0.000	*RARRES2*	-3.94	3.36	0.000
H19	6.67	8.47	0.000	*AQP1*	-3.91	4.05	0.000
MYOC	6.40	9.28	0.001	*SLC24A2*	-3.89	-1.78	0.025
HIF3A	6.19	2.63	0.000	*LAMP3*	-3.82	0.03	0.000
APOD	6.03	8.49	0.000	*GRIA2*	-3.82	0.29	0.001
APCDD1	5.95	2.15	0.000	*RBFOX1*	-3.80	-1.82	0.048
IGF2-AS	5.88	-1.83	0.002	*KRT17P1*	-3.77	-1.24	0.009
PNMT	5.76	-1.91	0.002	*TNFSF18*	-3.76	0.09	0.001
SAA1	5.75	1.16	0.000	*GRID2*	-3.67	-0.01	0.002
RAMP2-AS1	5.64	0.83	0.000	*VCAN-AS1*	-3.63	-1.95	0.003
ALOX15B	5.51	-0.80	0.000	*PADI2*	-3.55	0.96	0.000
ADH1B	5.38	6.06	0.000	*PI16*	-3.49	-0.82	0.008
PTGDR2	5.24	-2.22	0.003	*KLHDC7B*	-3.22	-0.85	0.003
LINC00525	5.13	-2.29	0.003	*LRRC15*	-3.21	4.55	0.017
FKBP5	4.87	7.62	0.000	*IGFL2*	-3.20	-2.15	0.040
LSP1	4.81	4.97	0.000	*C1orf87*	-3.10	-2.21	0.032
PRODH	4.58	2.59	0.000	*NPY6R*	-3.09	-2.22	0.036
P2RY14	4.57	0.98	0.000	*NMNAT2*	-3.02	3.71	0.014
STEAP4	4.57	3.40	0.000	*CLDN1*	-3.01	1.69	0.000
SCN3A	4.54	3.24	0.001	*ADM2*	-2.93	4.72	0.000
SFTPC	4.51	-2.59	0.021	*RIMS3*	-2.91	2.92	0.002
MAOA	4.44	4.35	0.000	*CCND2-AS1*	-2.91	-1.15	0.007
FAM107A	4.43	5.13	0.000	*IL32*	-2.91	2.34	0.007
HMGN2P15	4.37	-0.64	0.002	*TMEM63C*	-2.84	-1.45	0.003
ANGPTL7	4.31	9.66	0.007	*COL19A1*	-2.79	0.34	0.001
FHL5	4.24	-0.81	0.005	*NGEF*	-2.76	0.86	0.020
SOAT2	4.13	-0.86	0.001	*KAL1*	-2.75	2.99	0.007
PRR33	4.13	0.77	0.000	*WNT2*	-2.70	0.87	0.001
CPM	4.06	3.13	0.000	*VCAN*	-2.69	7.10	0.001
SAA2	4.06	-1.83	0.004	*CCND2-AS2*	-2.69	-1.72	0.032
TRAV39	4.03	-0.91	0.012	*C2orf40*	-2.67	1.60	0.000
IGF2	4.01	6.48	0.000	*NGFR*	-2.65	-1.76	0.020
LGI3	3.70	-0.07	0.001	*IPCEF1*	-2.64	-2.20	0.047
INHBB	3.67	1.41	0.000	*VNN1*	-2.63	-1.99	0.034
SAMHD1	3.57	7.45	0.000	*ARHGAP9*	-2.62	-0.48	0.003
RPA4	3.56	-2.16	0.006	*ADRA2A*	-2.60	2.91	0.001
PDK4	3.52	4.49	0.000	*RDH12*	-2.57	-1.62	0.012
ANKRD2	3.48	1.17	0.002	*PRSS35*	-2.57	3.69	0.001
LINC00702	3.44	4.81	0.000	*MYHAS*	-2.56	-1.50	0.012

ANGPTL1	3.42	2.28	0.000	*SLC14A1*	-2.53	0.08	0.047
CHI3L2	3.34	3.45	0.015	*EPHA3*	-2.52	0.55	0.007
SCARA5	3.34	-0.26	0.004	*CNGA1*	-2.49	-1.82	0.036
GPRC5B	3.26	5.02	0.000	*ELOVL2-AS1*	-2.46	-1.70	0.012
USP2	3.25	2.49	0.001	*KRT23*	-2.44	-1.92	0.036
TNNT3	3.12	-0.91	0.001	*RGS7BP*	-2.43	-0.27	0.031
POM121L9P	3.12	2.50	0.000	*CHAC1*	-2.43	4.19	0.000
ISM1	3.11	-0.30	0.001	*ATP1A3*	-2.43	-0.96	0.004
NCAM1-AS1	3.10	-2.39	0.046	*SEMA3D*	-2.42	1.49	0.026

Note: *Group #2:* DEGs between DEX and ETH treated GC-NR HTM cells

Supplementary Table S5c. List of Genes from Group #3

Gene	Group#1			Group#2		
	logFC	logCPM	*P* Value	logFC	logCPM	*P* Value
ZBTB16	5.60	4.66	0.000	8.48	4.59	0.000
OCA2	4.71	1.61	0.000	6.89	2.24	0.000
H19	4.44	7.60	0.000	6.67	8.47	0.000
MYOC	3.95	6.57	0.014	6.40	9.28	0.001
HIF3A	4.20	4.11	0.000	6.19	2.63	0.000
APOD	3.35	7.28	0.018	6.03	8.49	0.000
SAA1	6.34	2.81	0.000	5.75	1.16	0.000
ADH1B	3.39	7.72	0.001	5.38	6.06	0.000
FKBP5	3.99	7.23	0.000	4.87	7.62	0.000
LSP1	3.91	5.22	0.000	4.81	4.97	0.000
PRODH	4.46	3.17	0.000	4.58	2.59	0.000
P2RY14	4.14	1.13	0.001	4.57	0.98	0.000
SCN3A	3.09	0.86	0.008	4.54	3.24	0.001
MAOA	2.54	4.68	0.005	4.44	4.35	0.000
ANGPTL7	3.93	8.01	0.016	4.31	9.66	0.007
FHL5	2.99	-0.36	0.016	4.24	-0.81	0.005
PRR33	4.22	1.04	0.000	4.13	0.77	0.000
CPM	3.93	4.02	0.000	4.06	3.13	0.000
SAA2	4.71	0.71	0.003	4.06	-1.83	0.004
IGF2	3.56	6.66	0.000	4.01	6.48	0.000
SAMHD1	3.36	7.81	0.000	3.57	7.45	0.000
PDK4	2.24	5.55	0.000	3.52	4.49	0.000
LINC00702	3.82	3.76	0.000	3.44	4.81	0.000
ANGPTL1	3.54	3.59	0.000	3.42	2.28	0.000
USP2	2.22	2.21	0.015	3.25	2.49	0.001
MRO	2.68	1.62	0.037	3.10	1.99	0.003
AOX1	2.21	6.79	0.004	3.10	5.53	0.000
GALNT15	2.26	4.60	0.014	3.04	4.52	0.012
NEDD9	2.53	7.45	0.004	2.99	7.79	0.000
ACA59	2.04	-1.44	0.034	2.96	-1.20	0.007
ADH1A	2.85	1.95	0.014	2.95	0.45	0.000
FGFR4	2.50	3.10	0.029	2.92	1.76	0.001
ADRA1B	2.91	2.49	0.000	2.87	3.14	0.000
PPP1R14A	2.11	7.46	0.024	2.86	5.54	0.028
XRCC6P2	2.82	-0.36	0.001	2.84	-0.33	0.005
ABCA6	2.08	4.75	0.008	2.82	3.39	0.002
TIMP4	2.07	1.11	0.025	2.78	2.41	0.000
SLC16A12	3.72	1.63	0.000	2.75	-0.57	0.002
ADH4	2.54	-1.36	0.029	2.74	-0.95	0.005
FAM46B	2.15	4.36	0.001	2.53	4.96	0.001
SLC16A10	2.84	1.28	0.028	2.47	0.64	0.011

FPR1	2.70	-0.01	0.007	2.46	0.79	0.024
MOB3B	2.61	2.14	0.001	2.45	2.31	0.020
LINC01088	4.14	1.46	0.000	2.45	0.41	0.005
FMO2	3.38	3.38	0.002	2.40	1.29	0.002
TLDC2	3.48	1.28	0.000	2.38	1.05	0.003
ANGPTL5	2.39	0.70	0.015	2.25	1.24	0.001
OLAH	2.57	2.30	0.004	2.20	0.37	0.004
PER1	2.11	6.11	0.000	2.20	5.87	0.000
TRPV6	-3.05	1.87	0.019	2.17	0.67	0.014
DKK2	2.46	3.59	0.016	2.17	3.54	0.017
FST	-2.08	6.67	0.000	-2.01	6.50	0.000
TNFSF15	-2.05	0.31	0.017	-2.05	-1.40	0.026
CERS1	-2.05	-0.45	0.033	-2.07	-0.49	0.018
ARSI	-2.42	3.36	0.001	-2.13	3.10	0.000
TNNT2	-2.29	1.92	0.026	-2.14	-0.86	0.049
BST2	-4.64	1.92	0.000	-2.20	-0.98	0.012
RGS16	-2.34	5.10	0.007	-2.21	5.59	0.028
TAC3	-3.33	-1.20	0.002	-2.23	-1.29	0.033
FNDC1	-2.67	4.35	0.005	-2.27	5.07	0.003
SEMA6B	-2.30	1.97	0.016	-2.28	1.15	0.001
CPA4	-2.42	3.57	0.002	-2.34	2.68	0.034
CILP2	-2.02	0.07	0.026	-2.34	0.13	0.024
RGS7BP	-2.34	-1.10	0.029	-2.43	-0.27	0.031
KRT23	-3.53	0.24	0.023	-2.44	-1.92	0.036
SLC14A1	-2.58	2.94	0.041	-2.53	0.08	0.047
PRSS35	-2.32	3.30	0.000	-2.57	3.69	0.001
ADRA2A	-4.16	4.99	0.002	-2.60	2.91	0.001
NGFR	-2.06	0.83	0.041	-2.65	-1.76	0.020
C2orf40	-5.16	2.72	0.000	-2.67	1.60	0.000
WNT2	-3.86	2.59	0.000	-2.70	0.87	0.001
NGEF	-2.86	-0.57	0.016	-2.76	0.86	0.020
TMEM63C	-2.25	-1.54	0.042	-2.84	-1.45	0.003
LRRC15	-2.42	2.19	0.026	-3.21	4.55	0.017
PI16	-3.79	2.47	0.019	-3.49	-0.82	0.008
TNFSF18	-4.19	0.95	0.000	-3.76	0.09	0.001
LAMP3	-3.62	0.92	0.002	-3.82	0.03	0.000
AQP1	-6.33	7.61	0.000	-3.91	4.05	0.000
RARRES2	-2.19	5.97	0.016	-3.94	3.36	0.000
MXRA5Y	-2.78	-1.55	0.043	-5.08	-0.47	0.003

Note: *Group #3:* DEGs that are common to Group #1 and Group #2

Supplementary Table S5d: List of top 50 Up/Down-regulated Genes from Group #4

Up-regulated genes				Down-regulated genes			
**Gene**	**logFC**	**logCPM**	***P* Value**	**Gene**	**logFC**	**logCPM**	***P* Value**
FRG2C	5.27	-2.07	0.002	*UPK3A*	-8.49	0.36	0.001
NPSR1-AS1	5.07	-2.19	0.016	*RLN1*	-8.02	1.24	0.001
BHLHE22	5.04	0.77	0.012	*FAM110D*	-7.42	-0.59	0.002
SAA4	4.75	-2.34	0.028	*PRSS22*	-7.38	-0.62	0.002
TUSC5	3.79	-1.37	0.015	*NPY*	-7.26	3.41	0.000
LEP	3.75	-0.16	0.012	*GIMAP1*	-6.96	-0.98	0.001
RNA5SP111	3.63	-2.00	0.049	*ST14*	-6.81	1.52	0.001
KCNE1	3.55	0.74	0.010	*LRRC26*	-6.79	1.53	0.002
RPL7P57	3.49	-2.06	0.019	*KRT15*	-6.67	3.39	0.000
NTRK2	3.39	5.15	0.002	*FXYD3*	-6.59	2.02	0.001
GIP	3.36	-1.29	0.008	*PRAC1*	-6.58	-0.09	0.003
RN7SKP69	3.30	-1.68	0.004	*HOXB13*	-6.57	2.99	0.003
MIR5690	3.22	-1.75	0.012	*TPSB2*	-6.50	0.98	0.002
TNFAIP8L3	3.09	3.20	0.001	*ACPP*	-6.48	6.82	0.002
MAP1LC3C	3.01	3.54	0.026	*LCN2*	-6.47	0.44	0.001
SEMG1	2.97	-1.56	0.047	*KLK3*	-6.39	7.58	0.002
ABRA	2.93	0.31	0.012	*KCNH2*	-6.34	0.38	0.002
AFF2	2.90	1.40	0.007	*DOC2B*	-6.30	-0.33	0.001
BCRP1	2.82	-1.97	0.032	*LINC01297*	-6.30	1.17	0.002
HSPD1P11	2.81	1.79	0.001	*TPSAB1*	-6.26	0.71	0.001
C5AR2	2.76	0.11	0.006	*RBBP8NL*	-6.22	-1.54	0.009
MARCH10	2.75	-0.45	0.028	*CHRNA2*	-6.21	1.31	0.002
KRT18P62	2.74	-1.38	0.015	*SLC52A3*	-6.20	-0.35	0.002
HNRNPA3P11	2.73	-1.59	0.025	*KLK4*	-6.20	3.10	0.006
LINC00664	2.71	-2.02	0.035	*KRT5*	-6.19	2.48	0.001
KIAA1456	2.70	2.33	0.000	*MT1H*	-6.18	-1.57	0.005
B3GALT2	2.68	3.10	0.000	*KLK2*	-6.17	6.41	0.004
PLCE1-AS1	2.63	4.43	0.006	*CCDC64B*	-6.03	0.46	0.001
CYP51A1P2	2.62	-1.04	0.034	*SFN*	-6.02	1.83	0.000
RAPGEF5	2.62	3.56	0.010	*DES*	-5.98	6.03	0.001
CCDC54	2.60	-1.08	0.018	*MSMB*	-5.97	4.06	0.006
KLF15	2.58	4.75	0.000	*COL9A1*	-5.96	2.75	0.000
ITGA10	2.55	4.93	0.000	*C2orf54*	-5.94	-1.73	0.012
PDLIM1P4	2.54	-1.82	0.035	*PAGE4*	-5.94	0.47	0.004
CYP7B1	2.53	1.20	0.008	*EVX2*	-5.90	-1.76	0.008
GPX3	2.51	7.49	0.013	*PLVAP*	-5.87	1.64	0.002
ANO3	2.48	0.01	0.006	*RAMP3*	-5.83	0.30	0.002
NKAIN2	2.48	0.21	0.037	*RAB25*	-5.81	0.28	0.002
LINC00547	2.47	-0.51	0.013	*HOXA9*	-5.78	-0.78	0.007
ST7-AS2	2.44	-1.06	0.009	*CHRM1*	-5.77	0.88	0.003
FAM65C	2.38	3.31	0.012	*PDE9A*	-5.68	1.33	0.000

ACTG1P3	2.33	-1.48	0.038		*SHH*	-5.67	-1.91	0.010
C1QTNF7	2.30	3.78	0.000		*GIMAP7*	-5.67	-0.28	0.004
GJA5	2.26	0.01	0.049		*SPOCK3*	-5.64	1.74	0.001
FRMD3	2.21	1.81	0.006		*LCN6*	-5.61	-1.94	0.011
RN7SL608P	2.19	-1.55	0.028		*GNA15*	-5.60	-0.92	0.002
TSC22D3	2.19	8.12	0.000		*HOXA10*	-5.59	0.11	0.001
SLC38A11	2.16	2.84	0.014		*MMP7*	-5.58	1.84	0.004
LDHAL6B	2.15	-1.43	0.036		*GATA5*	-5.58	-0.87	0.009
FGF14	2.13	3.26	0.023		*LAD1*	-5.57	-0.94	0.006

Note: *Group #4:* Uniquely expressed DEGs of GC-R HTM cells (Group #1 minus Group #3)

Supplementary Table S5e: List of top 50 Up/Down-regulated Genes from Group #5

Up-regulated genes				Down-regulated genes			
**Gene**	**logFC**	**logCPM**	***P* Value**	**Gene**	**logFC**	**logCPM**	***P* Value**
RGCC	7.75	1.95	0.000	*GRM5*	-5.05	-2.43	0.010
APCDD1	5.95	2.15	0.000	*SLC24A2*	-3.89	-1.78	0.025
IGF2-AS	5.88	-1.83	0.002	*GRIA2*	-3.82	0.29	0.001
PNMT	5.76	-1.91	0.002	*RBFOX1*	-3.80	-1.82	0.048
RAMP2-AS1	5.64	0.83	0.000	*KRT17P1*	-3.77	-1.24	0.009
ALOX15B	5.51	-0.80	0.000	*GRID2*	-3.67	-0.01	0.002
PTGDR2	5.24	-2.22	0.003	*VCAN-AS1*	-3.63	-1.95	0.003
LINC00525	5.13	-2.29	0.003	*PADI2*	-3.55	0.96	0.000
STEAP4	4.57	3.40	0.000	*KLHDC7B*	-3.22	-0.85	0.003
SFTPC	4.51	-2.59	0.021	*IGFL2*	-3.20	-2.15	0.040
FAM107A	4.43	5.13	0.000	*C1orf87*	-3.10	-2.21	0.032
HMGN2P15	4.37	-0.64	0.002	*NPY6R*	-3.09	-2.22	0.036
SOAT2	4.13	-0.86	0.001	*NMNAT2*	-3.02	3.71	0.014
TRAV39	4.03	-0.91	0.012	*CLDN1*	-3.01	1.69	0.000
LGI3	3.70	-0.07	0.001	*ADM2*	-2.93	4.72	0.000
INHBB	3.67	1.41	0.000	*RIMS3*	-2.91	2.92	0.002
RPA4	3.56	-2.16	0.006	*CCND2-AS1*	-2.91	-1.15	0.007
ANKRD2	3.48	1.17	0.002	*IL32*	-2.91	2.34	0.007
CHI3L2	3.34	3.45	0.015	*COL19A1*	-2.79	0.34	0.001
SCARA5	3.34	-0.26	0.004	*KAL1*	-2.75	2.99	0.007
GPRC5B	3.26	5.02	0.000	*VCAN*	-2.69	7.10	0.001
TNNT3	3.12	-0.91	0.001	*CCND2-AS2*	-2.69	-1.72	0.032
POM121L9P	3.12	2.50	0.000	*IPCEF1*	-2.64	-2.20	0.047
ISM1	3.11	-0.30	0.001	*VNN1*	-2.63	-1.99	0.034
NCAM1-AS1	3.10	-2.39	0.046	*ARHGAP9*	-2.62	-0.48	0.003
STOX1	3.10	2.24	0.023	*RDH12*	-2.57	-1.62	0.012
MYBPHL	3.09	-2.40	0.040	*MYHAS*	-2.56	-1.50	0.012
C3	3.06	5.98	0.023	*EPHA3*	-2.52	0.55	0.007
LINC00704	3.04	-1.95	0.038	*CNGA1*	-2.49	-1.82	0.036
GGT5	3.04	6.46	0.000	*ELOVL2-AS1*	-2.46	-1.70	0.012
NR0B1	2.92	-1.01	0.016	*CHAC1*	-2.43	4.19	0.000
TRPC3	2.82	2.14	0.002	*ATP1A3*	-2.43	-0.96	0.004
FAM150B	2.80	-0.05	0.040	*SEMA3D*	-2.42	1.49	0.026
EDNRB	2.79	1.64	0.014	*CTNNA3*	-2.37	1.35	0.003
STAR	2.76	-1.13	0.002	*ERICH2*	-2.33	-1.49	0.036
WSCD1	2.74	0.98	0.034	*CCND2*	-2.32	2.26	0.019
RPL23AP81	2.74	-2.15	0.031	*LPHN3*	-2.30	2.21	0.043
ALDH1L1-AS2	2.64	-2.19	0.035	*INA*	-2.30	2.04	0.038
NRCAM	2.61	3.46	0.001	*UNC5B*	-2.28	5.69	0.009
AKR1B15	2.60	0.70	0.009	*CA3*	-2.27	-1.49	0.042
B3GNT7	2.60	1.89	0.009	*RIMS2*	-2.25	-1.14	0.045

SYN2	2.60	-0.79	0.002	*PLXNC1*	-2.23	2.68	0.003
PLIN5	2.59	-1.93	0.041	*LINC01133*	-2.21	2.67	0.003
SIX2	2.58	4.07	0.019	*LRRN4CL*	-2.19	2.88	0.005
MTSS1	2.51	4.67	0.008	*KCNS1*	-2.17	-0.42	0.010
DGKG	2.48	1.57	0.010	*KCTD16*	-2.17	2.93	0.002
MREG	2.47	2.69	0.001	*SLC7A5*	-2.14	6.66	0.004
SPP1	2.45	0.63	0.000	*KCNMB2*	-2.12	-1.25	0.033
HEYL	2.44	2.28	0.039	*BEX2*	-2.12	-0.68	0.018
TLE6	2.43	0.12	0.003	*CNTN6*	-2.11	1.58	0.010

Note: *Group #5:* Uniquely expressed DEGs of GC-NR HTM cells (Group #2 minus Group #3)

Supplementary Table S6a. List of Enriched Pathways in Group #1

Pathway	Fold Enrichment	P Value	Genes
Phosphatidylinositol signaling system	1.76	2E-03	*DGKG, PI4K2B, MTMR2, DGKE, DGKB, ITPR1, MTMR8, PIK3CD, ITPR2, PIK3CB, PIK3R1, PIK3C2A, MTMR6, MTMR7, MTM1, IMPA1, IMPA2, PPIP5K2, PIP4K2A, PLCE1, IP6K3, IPMK, INPP4B, PIKFYVE, PIK3CA, IMPAD1, PIK3C3, PLCB1, DGKI, DGKH*
Focal adhesion	1.73	7E-06	*ITGB1, GSK3B, FLT1, ITGB4, PIK3CD, PIK3CB, ARHGAP5, ACTB, IGF1R, PPP1CB, CCND3, AKT3, CAPN2, KDR, TNR, ITGAV, VAV3, PPP1R12A, ITGA4, ITGA2, ITGA1, ACTN4, PIK3CA, COL4A4, COL4A3, COL6A6, ITGA6, COL6A5, ITGA5, PPP1R12B, TLN1, MET, CRK, SOS2, BIRC2, SHC3, LAMA2, ROCK1, ROCK2, COL11A1, LAMA3, PDGFB, XIAP, PIK3R1, MYL12A, MYL12B, MAPK8, RAP1A, CHAD, SPP1, FLNA, MYL10, FLNB, PAK2, CAV2, CAV1, LAMB4, FN1, BRAF, PTK2, ITGA10, COL5A3*
TGF-beta signaling pathway	1.65	1E-02	*TGIF1, SMAD2, SMAD4, BMPR2, ROCK1, ZFYVE9, NOG, CUL1, BMP8A, INHBB, INHBA, GDF6, LTBP1, SMAD5, ACVR2B, TGFBR1, TGFBR2, BMP2, ZFYVE16, RBL1, RPS6KB1, MYC, BMPR1B, NODAL*
p53 signaling pathway	1.63	3E-02	*GADD45B, APAF1, SERPINE1, CCNB3, CCND3, SESN3, CCNE2, RRM2B, ZMAT3, CHEK2, CASP3, CCNG2, CCNG1, CHEK1, MDM2, FAS, MDM4, ATM, ATR*
mTOR signaling pathway	1.59	7E-02	*PRKAA1, PRKAA2, CAB39, STRADA, IRS1, PIK3CD, BRAF, TSC1, PIK3CB, PIK3R1, PIK3CA, RPS6KB1, RPS6KA2, DDIT4, AKT3, RICTOR*
Cell cycle	1.58	6E-03	*CDKN1C, RB1, GSK3B, CCNH, PRKDC, CUL1, SMC3, CDC14B, CCNB3, CCND3, CHEK2, MYC, ORC3, RAD21, ORC2, CHEK1, CDC27, BUB3, BUB1, SMAD2, SMAD4, GADD45B, RBL2, CCNA1, STAG1, WEE1, RBL1, DBF4, STAG2, CCNE2, MDM2, ANAPC4, ATM, ATR*
Adherens junction	1.54	6E-02	*SMAD2, SMAD4, YES1, INSR, ACTN4, SORBS1, IQGAP1, TGFBR1, ACTB, TGFBR2, IGF1R, TJP1, SNAI1, SNAI2, CTNNA2, WASF1, MAP3K7, MET, WASF3*
Regulation of actin cytoskeleton	1.51	1E-03	*ITGB1, CHRM2, NCKAP1, ITGB4, PIK3CD, PIK3CB, FGF1, FGF2, ACTB, FGF5, PPP1CB, CFL2, PIP4K2A, ITGAV, VAV3, PPP1R12A, ARHGEF12, ITGA4, ITGA2, ITGA1, ACTN4, ENAH, PIK3CA, ITGA6, ITGA5, PPP1R12B, CRK, SOS2, ARHGEF6, ROCK1, ROCK2, PDGFB, PIK3R1, IQGAP1, MYL12A, MYL12B, GNA13, NRAS, MYL10, WASF1, PAK2, FGF22, RDX, FN1, BRAF, ARPC5, SSH2, PTK2, PIKFYVE, DIAPH2, FGF14, APC, ITGA10, KRAS, FGFR4*
TNF signaling pathway	1.51	2E-02	*ATF2, CEBPB, TNFAIP3, PIK3CD, PIK3CB, PIK3R1, CXCL2, MAPK8, BAG4, CASP3, AKT3, MAP3K8, DNM1L, MAP3K7, MAP3K5, EDN1, CHUK, CFLAR, FOS, TNFRSF1B, NFKBIA, ITCH, PIK3CA, TRAF5, FAS, TAB3, TAB2, BIRC2*
MAPK signaling pathway	1.48	7E-04	*ATF2, ZAK, HSPB1, FGF1, FGF2, FGF5, MECOM, MYC, RPS6KA2, CASP3, AKT3, MAP3K8, MAP3K6, MAP3K7, MAP3K5, DUSP4, DUSP5, MEF2C, CHUK, DUSP1, CACNA2D3, CACNA2D4, FOS, TGFBR1, DUSP6, TGFBR2, CACNB2, PPM1A, PPM1B, TRAF6, RASA1, RASA2, RAPGEF2, CRK, SOS2, PDGFB, RASGRP2, RASGRP1, CACNG8, NRAS, PPP3R1, MAPK8, RAP1A, MAPK7, NTF3, FLNA, FLNB, CD14, PAK2, MAP4K3, FGF22, MAP3K2, NTRK2, GADD45B, NFATC3, BRAF, HSPA2, PPP5C, FGF14, TAOK1, NF1, FAS, KRAS, TAB2, FGFR4*
Pathways in cancer	1.32	3E-03	*RB1, ITGB1, GSK3B, WNT2B, HHIP, PIK3CD, CBLB, PIK3CB, FGF1, ETS1, FGF2, IGF1R, FGF5, EDNRB, MECOM, MYC, CASP3, AKT3, ITGAV, JAK1, APPL1, ARHGEF12, CHUK, ITGA2, WNT5A, MITF, FOS, TGFBR1, TGFBR2, MSH6, PIK3CA, CCNE2, MSH2, MSH3, COL4A4, TRAF6, COL4A3, TRAF5, AGTR1, RARB, ITGA6, PLCB1, MET, CRK, SOS2, BIRC2, LAMA2, ROCK1, ROCK2, CUL2, LAMA3, PTGER3, PDGFB, GNAI3, LPAR1, WNT8B, XIAP, PIK3R1, CBL, RASGRP2, RASGRP1, HIF1A, FOXO1, GNA13, NRAS, MAPK8, TPR, CTNNA2, FGF22, RUNX1T1, EGLN1, SMAD2, STAT5A, SMAD4, ZBTB16, PTCH1, FZD6, LAMB4, FN1, BRAF, PTK2, NFKBIA, BMP2, FGF14, APC, GNAQ, GNB4, MDM2, FAS, KRAS*

PI3K-Akt signaling pathway	1.30	9E-03	*ITGB1, CHRM2, ATF2, GSK3B, FLT1, IRS1, ITGB4, PIK3CD, BRCA1, PIK3CB, FGF1, FGF2, IGF1R, GHR, FGF5, CCND3, MYC, AKT3, KDR, TNR, ITGAV, JAK2, IL6R, JAK1, ITGA4, CHUK, ITGA2, ITGA1, TSC1, PRLR, RBL2, PIK3CA, CCNE2, COL4A4, COL4A3, DDIT4, MTCP1, COL6A6, ITGA6, SGK3, COL6A5, ITGA5, MET, TLR4, SOS2, TLR2, PHLPP2, PRKAA1, PRKAA2, LAMA2, COL11A1, LAMA3, PDGFB, LPAR1, PIK3R1, FOXO3, NRAS, BCL2L11, PPP2R3C, CHAD, SPP1, FGF22, MCL1, ANGPT1, INSR, LAMB4, FN1, PPP2R3A, PTK2, FGF14, RPS6KB1, ITGA10, COL5A3, GNB4, MDM2, KRAS, PKN2, FGFR4*
Cell adhesion molecules (CAMs)	-1.94	5E-10	*CD86, CNTNAP2, CNTNAP1, ITGAM, ITGB2, ICAM2, F11R, ITGAL, CLDN1, PTPRF, ICAM1, SPN, CDH5, CDH3, CDH1, ITGB7, HLA-DOA, NEO1, CD34, ICOSLG, HLA-DPA1, HLA-B, HLA-C, HLA-A, HLA-F, CLDN11, CLDN6, CLDN5, CLDN4, CLDN3, CD8A, CLDN8, CLDN7, PECAM1, CDH15, HLA-DQB1, ITGA9, NLGN3, SELPLG, NLGN2, SDC4, NRXN1, SDC2, SDC3, NRXN2, GLG1, HLA-DMA, HLA-DMB, LRRC4C, HLA-DQA2, CD99, HLA-DQA1, LRRC4B, CD276, NTNG2, HLA-DRB5, VCAM1, CADM1, SELE, SELP, CD2, CD4, OCLN, PTPRC, SELL, CD6, HLA-DPB1, HLA-DRA, CNTN2, ESAM, SIGLEC1, CD22, HLA-DRB1*
Calcium signaling pathway	-1.68	2E-07	*RYR1, CHRM3, RYR2, MYLK2, CHRM1, CALML6, HTR2B, ATP2A3, ADRA1D, CALML3, ATP2A1, GRPR, ADRA1A, SLC8A2, MYLK4, PPP3CA, CYSLTR1, CYSLTR2, BDKRB2, CD38, BDKRB1, NOS1, PRKACA, PDGFRA, PRKCB, TNNC1, TACR2, AVPR1A, TACR1, ITPKB, ADCY9, LTB4R2, ADORA2B, STIM2, ORAI2, SLC25A5, PLCB2, SLC25A6, CAMK2B, PDE1B, PTGER1, PDE1A, CHRNA7, CAMK2A, PTAFR, CACNA1B, ADCY4, CACNA1A, CACNA1D, ADRB1, ADCY2, ADCY1, CACNA1E, ADCY7, CACNA1G, GNA14, HRH1, GNA15, PLN, PHKG1, ERBB3, TBXA2R, PHKG2, ERBB2, PLCG2, DRD1, NTSR1, NOS3, ATP2B3, GRIN2C, GRIN1, P2RX7, P2RX6, P2RX4, P2RX2, P2RX1, CAMK4, CALM3, PLCD3, PLCD4*
Axon guidance	-1.63	6E-05	*ROBO3, RND1, PPP3CA, RGS3, NCK2, RAC2, RAC3, PLXNC1, EPHB2, EPHB1, EPHB4, EPHB3, SEMA6B, EPHA7, SEMA6A, UNC5B, UNC5D, RHOD, EPHA1, NGEF, EPHA3, EPHA2, SEMA3D, SEMA3A, SEMA3B, SEMA3G, CXCR4, SEMA3E, SEMA3F, EFNA4, NTN3, EFNB1, PAK1, EFNB3, ABL1, PAK6, PLXNA1, SLIT3, SLIT2, SRGAP3, PAK3, LRRC4C, PAK4, NTNG2, SEMA4A, SEMA4D, NFATC2, SEMA4F, SEMA4G, EFNA1, EFNA3, CXCL12, CDK5, FES, PLXNB2*
Inflammatory mediator regulation of TRP channels	-1.50	5E-03	*CAMK2B, CALML6, CAMK2A, ADCY4, HTR2B, PIK3R3, CALML3, ADCY2, PIK3R2, ADCY1, ADCY7, PIK3CG, PIK3R5, ADCY5, HRH1, PLCG2, BDKRB2, BDKRB1, TRPM8, PRKACA, ASIC1, CYP2J2, NTRK1, PLA2G4F, PRKCH, PRKCB, PRKCE, PRKCD, PLA2G4C, IGF1, NGF, MAPK12, MAPK11, ADCY9, TRPV4, IL1B, F2RL1, CALM3, PLCB2*
ECM-receptor interaction	-1.47	1E-02	*LAMA5, TNXB, SDC4, LAMC3, LAMA1, ITGA2B, TNC, LAMC2, THBS2, THBS4, THBS3, VTN, RELN, SV2B, SV2A, DAG1, ITGB7, ITGB6, CD36, LAMB3, COL24A1, VWF, COL1A1, COL3A1, COL1A2, COL4A2, COL5A1, ITGA11, COL4A6, COL6A3, ITGA7, AGRN, CD44, ITGA9*
Wnt signaling pathway	-1.39	7E-03	*FZD10, PPP3CA, FRAT2, CCND2, SOX17, RAC2, RAC3, PRKACA, MMP7, PRKCB, CTNNBIP1, WNT9A, WNT16, DKK1, SFRP4, SFRP1, SFRP2, PLCB2, TP53, CAMK2B, CTBP1, TCF7, CAMK2A, LEF1, LRP5, CXXC4, NKD2, sFRP2, WNT11, WIF1, DVL1, TBL1Y, DVL2, DVL3, GPC4, WNT2, WNT3, WNT4, FZD1, WNT10B, WNT10A, FZD2, WNT3A, FZD7, WNT7B, FZD9, FZD8, NFATC2, NFATC1, VANGL2, BAMBI*
cAMP signaling pathway	-1.33	5E-03	*RYR2, VIPR2, CHRM1, CALML6, PDE3B, CALML3, GLI1, PIK3CG, GLI3, CREB3L4, AKT2, CREB3L1, ADORA1, PDE4B, CNGA1, RAC2, RAC3, FFAR2, SOX9, PRKACA, PDE4C, NPY1R, SSTR1, ATP1B2, SSTR2, VAV1, VAV2, TIAM1, CREB3, ADCY9, RAPGEF3, CFTR, CREB5, CAMK2B, GRIA2, ADCYAP1R1, GIPR, NPR1, CAMK2A, ADCY4, PIK3R3, ATP1A3, CACNA1D, ADRB1, PIK3R2, ATP1A2, ADCY2, ADCY1, ADCY7, PIK3R5, ADCY5, PAK1, HCAR1, PLN, NPY, DRD1, GRIA4, BAD, BDNF, HTR1D, ATP2B3, NFATC1, GRIN2C, GRIN2B, TSHR, GRIN1, GRIN3B, CAMK4, FXYD2, CALM3*
Ras signaling pathway	-1.30	6E-03	*CALML6, FLT4, RASGRF2, RASGRF1, CALML3, PIK3CG, FGF7, SYNGAP1, FGF9, RASSF5, AKT2, RAC2, RAC3, PRKACA, PDGFRA, PLA2G4F, KSR1, PRKCB, HGF, PLA2G4C, NGF, PGF, PLA2G16, TIAM1,*

	MRAS, KIT, RIN1, PLA1A, EPHA2, SHC2, SHC1, PLA2G1B, PDGFA, PIK3R3, PIK3R2, PLA2G5, RASAL3, EFNA4, RASGRP4, PIK3R5, PAK1, GNG4, GNG7, PDGFC, ABL1, PLCG2, PAK6, PAK3, RALGDS, PAK4, ANGPT4, NGFR, ANGPT2, EGF, BAD, PLA2G2A, VEGFB, VEGFC, IGF1, GRIN2B, GNG11, GRIN1, VEGFA, EFNA1, FGF17, EFNA3, FGF18, CALM3, GNB5, RGL1, FGF13, FGF12, FGFR3, RGL2, FGF11, FGFR2, FGF10, FGFR1

Note: Group #1: The pathways enriched from DEGs between ETH and DEX-treated GC- R HTM cells; Size: Number of genes enriched. Fold enrichment is defined as the ratio of two proportions i.e. input genes/background information

Supplementary Table S6b. List of Enriched Pathways in Group #2.

Pathway	Fold Enrichment	P Value	Genes
TNF signaling pathway	1.65	2E-03	*CEBPB, TNFAIP3, PIK3R3, CXCL1, PIK3CB, PIK3R1, CXCL3, PTGS2, CXCL2, BAG4, CASP8, RPS6KA5, CASP10, CASP3, CCL2, MAPK1, RIPK1, MAP3K8, DNM1L, MAP3K5, JUN, EDN1, MLKL, CHUK, IL15, MMP3, CFLAR, FOS, TNFRSF1B, NFKBIA, ITCH, PIK3CA, IL1B, FAS, TAB3*
Cell cycle	1.50	9E-03	*CDKN1C, RB1, HDAC2, CDKN1B, PRKDC, CDC14A, ORC5, CCND3, PTTG1, YWHAQ, CHEK2, MYC, CDC26, RAD21, ORC2, CHEK1, E2F3, BUB3, SKP1, SMAD4, GADD45B, CDC25C, YWHAZ, GADD45G, RBL2, CCNA2, STAG1, WEE1, CDK7, RBL1, DBF4, CCNE2, CDK2, CDK1, MDM2, ANAPC4, MAD2L1*
Steroid biosynthesis	2.52	1E-02	*CYP27B1, EBP, SOAT1, SOAT2, CYP51A1, DHCR24, CEL, DHCR7, HSD17B7, LIPA*
MAPK signaling pathway	1.29	2E-02	*HSPB1, FGF1, RPS6KA6, RPS6KA5, MECOM, MYC, RPS6KA2, CASP3, MAP3K8, MAP3K6, MAP3K4, MAP3K5, DUSP4, PDGFRA, DUSP5, DUSP3, CHUK, DUSP1, IL1R1, PLA2G4B, RRAS2, PLA2G4A, PRKCA, FOS, MAPK8IP2, TGFBR1, DUSP6, TGFBR2, DUSP7, CACNB2, PPM1A, PPM1B, TRAF6, IL1B, RAPGEF2, RAF1, CRK, SOS2, RASGRP2, RASGRP4, CACNA1H, CACNA1G, CDC42, CACNG8, PPP3R1, RAP1A, MAPK7, FLNA, MAPK1, FLNB, MAP4K3, FGF22, NTRK2, JUN, GADD45B, BDNF, NFATC3, BRAF, HSPA2, GADD45G, PPP5C, FGF14, FAS, MAP3K13, FGFR4*
mTOR signaling pathway	1.65	3E-02	*PRKAA1, PRKAA2, CAB39, CAB39L, IRS1, RPS6, PIK3R3, BRAF, PRKCA, TSC1, PIK3CB, PIK3R1, MTOR, RPS6KA6, PIK3CA, RPS6KA2, ULK2, MAPK1, RICTOR*
Phosphatidylinositol signaling system	1.44	4E-02	*DGKG, PI4K2B, MTMR2, DGKB, ITPR1, MTMR8, PIK3R3, PIK3CB, PIK3R1, MTM1, IMPA1, IMPA2, PIP4K2A, PLCE1, PLCG1, IP6K3, IPMK, ITPK1, PRKCA, OCRL, INPP4B, SYNJ1, PIK3CA, IMPAD1, PIK3C3, CALM1, DGKI, DGKH*
Vascular smooth muscle contraction	1.38	5E-02	*GUCY1B3, RAMP2, ROCK1, ROCK2, NPR1, ITPR1, ADCY3, ADCY2, ADRA1B, ACTG2, PPP1CB, PPP1CC, KCNMB3, MAPK1, PPP1R14A, PTGIR, PLA2G4B, PLA2G4A, PRKCA, BRAF, ACTA2, ADORA2A, ADORA2B, GNAQ, KCNMA1, AGTR1, MRVI1, RAF1, CALM1, PPP1R12B, RAMP1, PPP1R12C*
VEGF signaling pathway	1.49	8E-02	*PLA2G4B, PXN, NFATC2, PLA2G4A, HSPB1, PIK3R3, PRKCA, PIK3CB, PIK3R1, PTGS2, PTK2, CDC42, PPP3R1, PIK3CA, KDR, MAPK1, PLCG1, RAF1*
TGF-beta signaling pathway	1.38	1E-01	*TGIF1, SMAD4, ROCK1, ZFYVE9, NOG, BMP8A, SMAD9, INHBB, GDF5, LTBP1, ACVR2B, DCN, TGFBR1, BMP6, TGFBR2, GDF7, PPP2CA, BMP2, RBL1, PPP2R1B, MYC, MAPK1, SKP1*
ECM-receptor interaction	-2.37	5E-09	*LAMA5, TNXB, LAMA2, SDC4, LAMA1, ITGA2B, TNC, LAMC2, THBS2, THBS4, THBS3, COMP, VTN, RELN, SV2A, DAG1, TNR, ITGB8, ITGB6, CD36, COL27A1, LAMB3, LAMB2, ITGA3, HSPG2, COL1A1, COL3A1, COL1A2, COL4A2, COL5A1, COL4A1, COL6A2, COL5A2, COL6A1, ITGA11, ITGA8, COL4A5, COL6A3, ITGA7, COL6A6, COL6A5, AGRN, CD44, ITGA9*
Axon guidance	-2.14	2E-09	*SEMA5A, ROBO3, LRRC4, RND1, RGS3, NCK2, RAC2, RAC3, PLXNC1, EPHB2, HRAS, EPHB1, EPHB4, EPHB3, SEMA6B, EPHA5, UNC5B, EPHA8, UNC5C, UNC5D, RHOD, RASA1, MET, NGEF, EPHA3, EPHA2, SEMA3D, SEMA3B, NTN4, SEMA3E, SEMA3F, EFNA4, NTN3, GNAI2, EFNB2, EFNB1, PAK1, EFNB3, ABL1, PAK6, PLXNA1, SLIT3, FYN, SRGAP3, PAK3, SRGAP2, LRRC4C, NTNG1, NTNG2, SEMA4A, LIMK1, SEMA4F, SEMA4G, L1CAM, EFNA3, CXCL12, PLXNB3, PLXNB2*
ABC transporters	-1.81	2E-02	*ABCD4, ABCA2, ABCD2, ABCB1, ABCA3, ABCB4, TAP2, TAP1, ABCA7, ABCC9, ABCB9, ABCB11, ABCA12, ABCC11, ABCA13, ABCG1, CFTR*
Notch signaling pathway	-1.75	2E-02	*JAG2, NUMBL, JAG1, CTBP1, PSEN2, DTX1, DTX2, RFNG, DLL1, DLL3, APH1A, DLL4, LFNG, NCSTN, DVL1, DVL2, DVL3, HES5*

Cell adhesion molecules (CAMs)	-1.71	4E-05	*CNTNAP2, CNTNAP1, ITGB2, ICAM2, LRRC4, ITGAL, PVR, CLDN1, PTPRF, ICAM1, CDH5, CDH3, CDH2, CDH1, MPZ, ITGB8, NEO1, CD34, HLA-B, HLA-C, HLA-A, HLA-F, VCAN, CLDN7, ITGA8, CDH15, ITGA9, NLGN3, SELPLG, NLGN2, SDC4, NRXN1, SDC2, SDC3, NRXN2, GLG1, HLA-DMA, LRRC4C, CD99, JAM2, LRRC4B, CD276, NTNG1, NTNG2, CADM3, VCAM1, CADM1, L1CAM, CD4, OCLN, PTPRC, SIGLEC1*
Gap junction	-1.65	4E-03	*ADCY4, HTR2B, HTR2A, ADCY1, ADCY8, ADCY7, GRM1, GNAI2, ADCY5, TUBA1C, TUBB6, GRM5, TUBA1A, TUBB3, PDGFC, PRKACA, HRAS, PRKG1, MAP2K5, GUCY1A3, GUCY1A2, MAP2K1, PRKCB, EGF, TUBB, PLCB4, ADCY9, GNAS, GRB2, SOS1, PLCB2*
Inflammatory mediator regulation of TRP channels	-1.57	6E-03	*CALML6, CAMK2A, ADCY4, HTR2B, PIK3R2, HTR2A, ADCY1, ADCY8, ADCY7, ADCY5, PLCG2, BDKRB2, BDKRB1, PRKACA, MAP2K6, ASIC1, MAP2K3, NTRK1, PRKCH, PRKCB, PRKCE, PLA2G4C, IGF1, NGF, MAPK10, MAPK11, PLCB4, ADCY9, TRPV4, GNAS, PRKCQ, PLCB2, CALM2*
Long-term depression	-1.48	7E-02	*GUCY1A3, GUCY1A2, GRIA2, GNAZ, MAP2K1, GRID2, PRKCB, PLA2G4C, CACNA1A, IGF1, GRM1, GNAI2, PLCB4, GNA12, GNAS, HRAS, PLCB2, GRIA3, PRKG1*
Ras signaling pathway	-1.43	1E-03	*CALML6, RASGRF2, ELK1, FGF7, RASSF5, RAC2, RAC3, PRKACA, HRAS, MAP2K1, KSR1, PRKCB, HGF, PLA2G4C, NGF, PLA2G16, TIAM1, MRAS, RASA1, KIT, SOS1, MET, PLA1A, EPHA2, SHC4, SHC2, SHC1, PLA2G1B, PIK3R2, PLD1, RASGRP1, EFNA4, RELA, RASGRP3, PAK1, GNG4, GNG7, PDGFC, ABL1, PLCG2, PAK6, PAK3, RALGDS, ANGPT4, NGFR, ANGPT2, EGF, BAD, VEGFB, VEGFC, IGF1, GRIN2B, GNG11, NFKB1, VEGFA, MAPK10, FGF17, EFNA3, KITLG, FGF18, GRB2, GNB5, TEK, CALM2, FGF12, FGFR3, RGL2, FGFR2, FGFR1*
PI3K-Akt signaling pathway	-1.42	5E-05	*EPO, ITGA2B, TNC, COMP, STK11, FGF7, CCND2, CCND1, CREB3L4, CREB3L1, TNR, IFNAR2, MAP2K1, COL27A1, IL4R, HGF, F2R, COL4A2, COL4A1, DDIT4, COL4A5, MTCP1, TP53, EPHA2, ATF4, PIK3R2, EFNA4, VTN, PDGFC, EIF4EBP1, PCK2, NGFR, IGF1, GNG11, NFKB1, FGF17, COL1A1, EFNA3, IL6, CDK6, COL1A2, COL5A1, IL7, CDK4, COL5A2, ITGA11, FGF18, GRB2, GNB5, IL7R, FGF12, FGFR3, FGFR2, FGFR1, CDKN1A, LAMC2, CASP9, MLST8, ITGB8, ITGB6, JAK3, HRAS, ITGA3, NGF, CREB3, COL6A2, COL6A1, KIT, ITGA8, COL6A3, ITGA7, COL6A6, COL6A5, SOS1, MET, ITGA9, CREB5, LAMA5, PHLPP1, TNXB, LAMA2, ATF6B, LAMA1, THBS2, RELA, THBS4, THBS3, RELN, GNG4, GNG7, ANGPT4, ANGPT2, LAMB3, BAD, LAMB2, EGF, VEGFB, VEGFC, PPP2R3B, VEGFA, COL3A1, KITLG, TEK, PKN1, PIK3AP1*
cGMP-PKG signaling pathway	-1.42	8E-03	*ATF6B, CALML6, ADCY4, ATP1A4, ATP2A3, ATP1A3, ATP1A2, ADRB2, ADCY1, ADCY8, ADCY7, SLC8A1, GNAI2, ADCY5, CREB3L4, CREB3L1, KCNMB2, GNA12, ADORA1, CNGA1, BDKRB2, KCNMB4, CACNA1S, PRKG1, GUCY1A3, GUCY1A2, MAP2K1, MEF2C, KCNJ8, BAD, PRKCE, PDE2A, ATP2B3, ATP2B1, ADRA2B, ADRA2A, CREB3, KCNU1, PLCB4, ADCY9, PDE3A, PDE5A, SLC25A5, PLCB2, CALM2, ATF4, SLC25A6, CREB5*
Calcium signaling pathway	-1.38	9E-03	*CHRM3, RYR2, CALML6, CHRM5, HTR2B, ATP2A3, HTR2A, HTR4, SLC8A1, GRM1, GRM5, CYSLTR2, BDKRB2, BDKRB1, PRKACA, PRKCB, TNNC1, TNNC2, F2R, TACR1, ITPKB, PLCB4, ADCY9, ITPKA, ORAI2, SLC25A5, PLCB2, SLC25A6, PTGER1, PDE1A, CAMK2A, ADCY4, CACNA1A, ADRB2, ADCY1, ADCY8, CACNA1E, ADCY7, GNA14, PHKG1, ERBB3, TBXA2R, PLCG2, CACNA1S, ATP2B3, ATP2B1, P2RX7, P2RX6, P2RX4, GNAS, PLCD3, CALM2, PLCD4*
Regulation of actin cytoskeleton	-1.25	5E-02	*CYFIP2, CHRM3, ARPC1B, ITGA2B, ITGB2, CHRM5, ARPC5L, ITGAL, FGF7, BDKRB2, RAC2, BDKRB1, ITGB8, RAC3, PIP4K2B, ITGB6, HRAS, APC2, MAP2K1, ITGA3, INSRR, F2R, VAV2, TIAM1, MRAS, ITGA8, PFN4, ARHGEF4, ITGA7, SOS1, ITGA9, PFN2, PIK3R2, IQGAP2, IQGAP3, FGD1, PAK1, GNA12, PDGFC, PIP5K1A, PAK6, NCKAP1L, PAK3, EGF, LIMK1, BAIAP2, SSH3, MYLPF, FGF17, DIAPH3, ITGA11, FGF18, FGF12, FGFR3, FGFR2, FGFR1*

Note: Group #2: The pathways Enriched from DEGs between ETH and DEX-treated GC- NR HTM cells; Size: Number of genes enriched.

Supplementary Table S6c. List of Enriched Pathways in Group #3.

Pathway	Fold Enrichment	P Value	Genes
Focal adhesion	1.86	1E-04	*FLT1, SHC3, ROCK1, ROCK2, ITGB4, COL11A1, LAMA3, XIAP, PIK3CB, PIK3R1, MYL12A, ACTB, MYL12B, PPP1CB, CCND3, RAP1A, KDR, SPP1, CAPN2, FLNA, MYL10, FLNB, VAV3, ITGA4, CAV2, CAV1, ITGA2, LAMB4, BRAF, PTK2, PIK3CA, ITGA10, COL4A4, COL4A3, ITGA6, ITGA5, PPP1R12B, TLN1, CRK, SOS2*
Phosphatidylinositol signaling system	2.05	2E-03	*DGKG, PI4K2B, MTMR2, DGKB, IPMK, ITPR1, MTMR8, PIK3CB, PIK3R1, MTM1, INPP4B, PIK3CA, IMPA1, IMPA2, IMPAD1, PIP4K2A, PLCE1, PIK3C3, IP6K3, DGKI, DGKH*
MAPK signaling pathway	1.59	2E-03	*HSPB1, FGF1, RASGRP2, CACNG8, PPP3R1, RAP1A, MAPK7, MECOM, MYC, CASP3, RPS6KA2, FLNA, FLNB, MAP3K8, MAP3K6, MAP4K3, FGF22, MAP3K5, DUSP4, NTRK2, DUSP5, GADD45B, CHUK, DUSP1, NFATC3, BRAF, HSPA2, FOS, TGFBR1, DUSP6, TGFBR2, CACNB2, PPM1A, PPP5C, PPM1B, FGF14, TRAF6, RAPGEF2, FAS, FGFR4, CRK, SOS2*
Pathways in cancer	1.39	8E-02	*RB1, WNT2B, PIK3CB, FGF1, ETS1, EDNRB, MECOM, MYC, CASP3, JAK1, CHUK, ITGA2, WNT5A, MITF, FOS, TGFBR1, TGFBR2, MSH6, PIK3CA, CCNE2, COL4A4, TRAF6, COL4A3, AGTR1, RARB, ITGA6, CRK, SOS2, ROCK1, ROCK2, LAMA3, GNAI3, LPAR1, WNT8B, XIAP, PIK3R1, CBL, RASGRP2, HIF1A, FOXO1, FGF22, STAT5A, SMAD4, ZBTB16, PTCH1, FZD6, LAMB4, BRAF, PTK2, NFKBIA, BMP2, FGF14, APC, GNAQ, GNB4, MDM2, FAS*
TNF signaling pathway	1.79	1E-02	*EDN1, CEBPB, CHUK, TNFAIP3, CFLAR, PIK3CB, PIK3R1, FOS, TNFRSF1B, CXCL2, NFKBIA, ITCH, BAG4, PIK3CA, CASP3, FAS, TAB3, MAP3K8, DNM1L, MAP3K5*
p53 signaling pathway	2.00	2E-02	*GADD45B, APAF1, SERPINE1, CCND3, SESN3, CCNE2, CHEK2, CASP3, CCNG2, CCNG1, CHEK1, MDM2, FAS, MDM4*
Drug metabolism - cytochrome P450	1.97	2E-02	*MAOA, ADH1B, ADH1A, MGST1, FMO2, FMO3, FMO4, FMO5, ADH5, ADH6, ADH4, CYP2A6, ALDH3B1, AOX1*
Adipocytokine signaling pathway	1.91	3E-02	*PRKAA1, PRKAB2, PRKAA2, CHUK, ACSL1, IRS1, PRKAG2, IRS2, PTPN11, TNFRSF1B, NFKBIA, ACSBG1, JAK2, PPARA*
Regulation of actin cytoskeleton	1.46	3E-02	*ROCK1, ROCK2, ITGB4, PIK3CB, PIK3R1, FGF1, MYL12A, ACTB, MYL12B, PPP1CB, MYL10, PIP4K2A, FGF22, VAV3, ITGA4, ITGA2, BRAF, ARPC5, SSH2, PTK2, ENAH, DIAPH2, FGF14, APC, PIK3CA, ITGA10, ITGA6, ITGA5, FGFR4, PPP1R12B, CRK, SOS2*
Thyroid hormone signaling pathway	1.58	5E-02	*NCOA2, MED1, NCOA3, SLC16A10, PIK3CB, PIK3R1, ATP1B1, ESR1, HIF1A, FOXO1, ACTB, MED13L, MED14, NCOR1, PIK3CA, TBC1D4, MYC, MDM2, PLCE1*
Cell cycle	1.54	5E-02	*CDKN1C, RB1, SMAD4, GADD45B, PRKDC, RBL2, CCND3, STAG1, WEE1, RBL1, DBF4, CCNE2, CHEK2, MYC, RAD21, ORC2, CHEK1, MDM2, ANAPC4, BUB3*
mTOR signaling pathway	1.81	8E-02	*PRKAA1, PRKAA2, CAB39, PIK3CA, IRS1, RPS6KA2, TSC1, BRAF, RICTOR, PIK3CB, PIK3R1*
PI3K-Akt signaling pathway	1.25	9E-02	*PRKAA1, FLT1, PRKAA2, IRS1, ITGB4, COL11A1, LAMA3, LPAR1, BRCA1, PIK3CB, PIK3R1, FOXO3, FGF1, CCND3, BCL2L11, MYC, KDR, SPP1, JAK2, IL6R, FGF22, JAK1, MCL1, ANGPT1, ITGA4, CHUK, ITGA2, INSR, LAMB4, TSC1, PRLR, PTK2, RBL2, FGF14, PIK3CA, CCNE2, ITGA10, COL4A4, COL4A3, GNB4, MDM2, ITGA6, ITGA5, FGFR4, SOS2*
TGF-beta signaling pathway	1.59	9E-02	*TGIF1, SMAD4, ROCK1, ZFYVE9, NOG, BMP8A, INHBB, LTBP1, ACVR2B, TGFBR1, TGFBR2, BMP2, RBL1, MYC*
Axon guidance	-2.25	5E-07	*ROBO3, SEMA3D, SEMA3B, SEMA3E, SEMA3F, EFNA4, RND1, NTN3, EFNB1, PAK1, EFNB3, RGS3, ABL1, NCK2, RAC2, PAK6, PLXNA1, RAC3, SLIT3, PLXNC1, SRGAP3, EPHB2, PAK3, LRRC4C, EPHB1, EPHB4, EPHB3, SEMA6B, NTNG2, SEMA4A, UNC5B, SEMA4F, UNC5D, SEMA4G, RHOD, EFNA3, CXCL12, PLXNB2, EPHA3, NGEF, EPHA2*

Cell adhesion molecules (CAMs)	-2.01	1E-05	*NLGN3, CNTNAP2, CNTNAP1, SELPLG, NLGN2, SDC4, NRXN1, SDC2, SDC3, ITGB2, ICAM2, NRXN2, ITGAL, CLDN1, PTPRF, GLG1, ICAM1, CDH5, HLA-DMA, CDH3, CDH1, LRRC4C, NEO1, CD34, CD99, LRRC4B, CD276, NTNG2, VCAM1, CADM1, HLA-B, HLA-C, HLA-A, HLA-F, CD4, OCLN, PTPRC, CLDN7, SIGLEC1, CDH15, ITGA9*
ECM-receptor interaction	-2.24	5E-05	*LAMA5, TNXB, SDC4, LAMA1, ITGA2B, TNC, LAMC2, THBS2, THBS4, THBS3, VTN, RELN, SV2A, DAG1, ITGB6, CD36, LAMB3, COL1A1, COL3A1, COL1A2, COL4A2, COL5A1, ITGA11, COL6A3, ITGA7, AGRN, CD44, ITGA9*
Ras signaling pathway	-1.60	5E-04	*CALML6, RASGRF2, FGF7, RASSF5, RAC2, RAC3, PRKACA, KSR1, PRKCB, HGF, PLA2G4C, NGF, PLA2G16, TIAM1, MRAS, KIT, PLA1A, EPHA2, SHC2, SHC1, PLA2G1B, PIK3R2, EFNA4, PAK1, GNG4, GNG7, PDGFC, ABL1, PLCG2, PAK6, PAK3, RALGDS, ANGPT4, NGFR, ANGPT2, EGF, BAD, VEGFB, VEGFC, IGF1, GRIN2B, GNG11, VEGFA, FGF17, EFNA3, FGF18, GNB5, FGF12, FGFR3, RGL2, FGFR2, FGFR1*
Aldosterone synthesis and secretion	-1.98	2E-03	*SCARB1, DAGLA, CAMK1D, CALML6, PRKCB, PRKCE, CAMK2A, PDE2A, ADCY4, ADCY1, ADCY7, ADCY5, CREB3, ADCY9, CREB3L4, CYP11A1, CREB3L1, CAMK1, PRKACA, PLCB2, CAMK1G, CREB5, ATF4*
Thyroid hormone synthesis	-1.99	4E-03	*GPX1, HSPA5, PRKCB, ADCY4, GPX7, ATP1A3, ATP1A2, ADCY1, ADCY7, TSHR, PDIA4, ADCY5, CREB3, ADCY9, CREB3L4, CREB3L1, PRKACA, PLCB2, CREB5, ATF4*
Inflammatory mediator regulation of TRP channels	-1.70	1E-02	*NTRK1, PRKCH, CALML6, PRKCB, PRKCE, CAMK2A, PLA2G4C, ADCY4, HTR2B, PIK3R2, ADCY1, IGF1, NGF, ADCY7, ADCY5, MAPK11, ADCY9, TRPV4, BDKRB2, PLCG2, BDKRB1, PRKACA, PLCB2, ASIC1*
Notch signaling pathway	-2.03	2E-02	*JAG2, CTBP1, PSEN2, DTX1, DTX2, RFNG, DLL1, DLL3, APH1A, DLL4, LFNG, DVL1, DVL2, DVL3*
Glycine, serine and threonine metabolism	-2.14	2E-02	*GAMT, AOC3, AOC2, PSAT1, SHMT2, CHDH, PGAM1, SARDH, PHGDH, SDSL, ALDH7A1, PSPH*
Calcium signaling pathway	-1.44	2E-02	*CHRM3, RYR2, PTGER1, CALML6, PDE1A, CAMK2A, ADCY4, HTR2B, ATP2A3, CACNA1A, ADCY1, CACNA1E, ADCY7, GNA14, CYSLTR2, PHKG1, ERBB3, TBXA2R, PLCG2, BDKRB2, BDKRB1, PRKACA, PRKCB, TNNC1, ATP2B3, TACR1, P2RX7, ITPKB, P2RX6, P2RX4, ADCY9, ORAI2, PLCD3, SLC25A5, PLCD4, PLCB2, SLC25A6*
Protein processing in endoplasmic reticulum	-1.44	2E-02	*RPN2, PRKCSH, RPN1, RRBP1, HERPUD1, SEC61A1, GANAB, OS9, LMAN2, MAN1C1, FBXO6, BAK1, SIL1, UBQLN4, TXNDC5, PDIA3, SSR4, HSPA5, WFS1, SSR2, FBXO2, HSPA6, UBE2E2, TRAF2, CKAP4, DDOST, PDIA4, DNAJC5, DDIT3, ERP29, HYOU1, P4HB, CALR, MAN1B1, ATF4*
Wnt signaling pathway	-1.46	3E-02	*CTBP1, TCF7, LEF1, CAMK2A, LRP5, FZD10, FRAT2, WNT11, CCND2, DVL1, DVL2, RAC2, DVL3, RAC3, PRKACA, WNT2, WNT3, WNT4, WNT10B, FZD2, PRKCB, FZD7, CTNNBIP1, FZD8, WNT9A, SFRP4, VANGL2, PLCB2, TP53*
ABC transporters	-1.74	9E-02	*ABCA2, ABCB1, ABCA3, ABCB4, TAP2, TAP1, ABCA7, ABCB9, ABCC11, ABCG1, CFTR*

Note: Group #3: The pathways Enriched from the overlapping DEGs between Group #1 and Group #2; Size: Number of genes enriched.

Supplementary Table S6d. List of Enriched Pathways in Group #4.

Pathway	Fold Enrichment	P Value	Genes
Axon guidance	1.94	1E-02	*ITGB1, GSK3B, SEMA7A, ARHGEF12, SEMA3C, LRRC4, NTN4, UNC5C, NRAS, RASA1, DPYSL2, CFL2, KRAS, PAK2, MET, SRGAP1, NCK1*
Regulation of actin cytoskeleton	1.59	3E-02	*ITGB1, CHRM2, NCKAP1, ARHGEF12, PPP1R12A, RDX, ITGA1, PDGFB, FN1, PIK3CD, ACTN4, IQGAP1, FGF2, FGF5, GNA13, PIKFYVE, NRAS, CFL2, ITGAV, KRAS, WASF1, PAK2, ARHGEF6*
ECM-receptor interaction	2.00	3E-02	*ITGB1, LAMA2, COL5A3, ITGA1, CHAD, FN1, TNR, SDC1, COL6A6, ITGAV, GP1BA, COL6A5*
Focal adhesion	1.55	5E-02	*ITGB1, GSK3B, PPP1R12A, LAMA2, ITGA1, PDGFB, FN1, PIK3CD, ACTN4, ARHGAP5, IGF1R, MAPK8, COL5A3, CHAD, AKT3, TNR, COL6A6, ITGAV, COL6A5, PAK2, MET, BIRC2*
PI3K-Akt signaling pathway	1.39	5E-02	*ITGB1, CHRM2, PHLPP2, ATF2, GSK3B, LAMA2, PDGFB, PIK3CD, FGF2, IGF1R, GHR, FGF5, NRAS, PPP2R3C, CHAD, AKT3, TNR, ITGAV, ITGA1, FN1, PPP2R3A, RPS6KB1, COL5A3, DDIT4, MTCP1, KRAS, PKN2, COL6A6, SGK3, COL6A5, MET, TLR4, TLR2*
Adherens junction	2.04	5E-02	*SMAD2, TJP1, YES1, ACTN4, CTNNA2, IQGAP1, WASF1, MAP3K7, MET, IGF1R*
Protein processing in endoplasmic reticulum	1.54	7E-02	*SEC23A, SEC24A, SAR1B, SEL1L, CUL1, EIF2AK2, YOD1, EIF2S1, UBE2J1, DNAJC3, MAPK8, HSPH1, MAN1A2, MAN1A1, UGGT2, SEC62, ATF6, MBTPS2*
T cell receptor signaling pathway	1.74	8E-02	*GSK3B, DLG1, NRAS, AKT3, PIK3CD, KRAS, MAP3K7, PAK2, RASGRP1, LAT, MALT1, NCK1*
Cell cycle	1.64	8E-02	*SMAD2, GSK3B, CCNH, CUL1, SMC3, CDC14B, CCNB3, CCNA1, STAG2, ORC3, CDC27, ATM, BUB1, ATR*
Calcium signaling pathway	-1.98	1E-05	*CAMK2B, RYR1, MYLK2, CHRM1, PDE1B, CHRNA7, PTAFR, CACNA1B, CACNA1D, ADRA1D, ADRB1, ATP2A1, CALML3, ADCY2, GRPR, ADRA1A, SLC8A2, CACNA1G, MYLK4, PPP3CA, HRH1, GNA15, CYSLTR1, PLN, PHKG2, ERBB2, CD38, DRD1, NOS1, NTSR1, PDGFRA, NOS3, TACR2, AVPR1A, GRIN2C, GRIN1, LTB4R2, ADORA2B, STIM2, P2RX2, P2RX1, CAMK4, CALM3*
Type I diabetes mellitus	-3.33	1E-05	*CD86, HLA-DRB5, PTPRN2, PTPRN, GAD1, PRF1, HLA-DMB, IL1B, HLA-DPB1, HLA-DRA, IL12A, HLA-DOA, HLA-DQA2, HLA-DQA1, HLA-DRB1, HLA-DPA1, HLA-DQB1*
Cell adhesion molecules (CAMs)	-1.85	7E-04	*CD86, ITGAM, F11R, SPN, HLA-DMB, ITGB7, HLA-DOA, HLA-DQA2, ICOSLG, HLA-DQA1, HLA-DPA1, HLA-DRB5, SELE, SELP, CLDN11, CD2, CLDN6, CLDN5, CLDN4, CLDN3, SELL, CD6, CD8A, CLDN8, HLA-DPB1, PECAM1, HLA-DRA, CNTN2, ESAM, CD22, HLA-DRB1, HLA-DQB1*
B cell receptor signaling pathway	-2.03	7E-03	*LYN, SYK, NFATC2, PIK3R3, NFATC1, PIK3CG, VAV1, PIK3R5, CD79B, CD79A, PPP3CA, AKT2, BLNK, PTPN6, FCGR2B, CD22, CARD11*
TNF signaling pathway	-1.69	2E-02	*MLKL, RIPK3, IL15, MMP3, LIF, PIK3R3, CXCL1, CXCL3, SELE, MMP9, PIK3CG, MAPK12, PIK3R5, TNFRSF1A, CXCL10, SOCS3, CASP10, IL1B, AKT2, CCL5, CCL2, PGAM5*
Chemokine signaling pathway	-1.37	6E-02	*CCL14, CCL13, CXCL6, GSK3A, CXCL9, NCF1, WAS, CXCR4, PIK3R3, CXCL1, ADCY2, ARRB2, CXCL13, CXCL3, CXCL14, PIK3CG, PIK3R5, PREX1, AKT2, CCL5, CCL2, CCL19, CCR1, LYN, PRKCD, VAV1, FGR, CXCL10, HCK, CXCL11, CCL26*

Note: Group #4: Uniquely expressed GC-R HTM cells specific pathways enriched from DEGs of Group #3 minus Group #1; Size: Number of genes enriched.

Supplementary Table S6e. List of Enriched Pathways in Group #5.

Pathway	Fold Enrichment	P Value	Genes
Long-term depression	2.31	9E-03	*RYR1, GUCY1B3, PLA2G4B, PLA2G4A, PRKCA, GNAI1, PPP2CA, GNAO1, PPP2R1B, PRKG2, MAPK1, NOS1, RAF1*
Vascular smooth muscle contraction	1.82	1E-02	*GUCY1B3, PTGIR, RAMP2, NPR1, PLA2G4B, PLA2G4A, ADCY3, PRKCA, ADCY2, ACTG2, PPP1CC, ADORA2A, ADORA2B, KCNMA1, MAPK1, MRVI1, CALM1, RAF1, PPP1R12C, RAMP1*
Inflammatory mediator regulation of TRP channels	1.85	2E-02	*PTGER4, IL1R1, PTGER2, PLA2G4B, PLA2G4A, ADCY3, TRPV3, PIK3R3, PRKCA, ADCY2, ALOX12, PPP1CC, IL1B, F2RL1, PLCG1, CALM1, ASIC3*
Fatty acid metabolism	2.22	3E-02	*CPT1A, FASN, OXSM, ACSL6, ACSL5, ELOVL6, HADH, CPT1B, ACAT2, ACAT1*
VEGF signaling pathway	1.92	6E-02	*CDC42, PLA2G4B, PXN, NFATC2, PIK3R3, PLA2G4A, MAPK1, PRKCA, PLCG1, RAF1, PTGS2*
Oxytocin signaling pathway	1.49	7E-02	*RYR1, GUCY1B3, JUN, NPR1, PLA2G4B, KCNJ14, NFATC2, PLA2G4A, ADCY3, PRKCA, ADCY2, PTGS2, GNAI1, GNAO1, PPP1CC, RGS2, CAMK4, MAPK1, CALM1, RAF1, PPP1R12C*
Calcium signaling pathway	1.43	7E-02	*RYR1, PDGFRA, PTGFR, PDE1C, ATP2A2, ADCY3, ATP2A1, PRKCA, ADCY2, GRIN2C, CACNA1H, SLC8A2, CACNA1G, PLN, GNAL, STIM1, ADORA2A, ADORA2B, CAMK4, P2RX1, VDAC3, NOS1, PLCG1, CALM1*
ECM-receptor interaction	-2.62	9E-04	*COL27A1, LAMA2, LAMB2, ITGA3, HSPG2, COMP, COL4A1, COL6A2, COL5A2, COL6A1, ITGA8, COL4A5, TNR, ITGB8, COL6A6, COL6A5*
Focal adhesion	-1.94	1E-03	*SHC4, LAMA2, ELK1, COMP, CCND1, TNR, ITGB8, FYN, HRAS, MAP2K1, COL27A1, LAMB2, ITGA3, PARVA, MYLPF, MAPK10, COL4A1, COL6A2, COL5A2, COL6A1, ITGA8, COL4A5, GRB2, COL6A6, TLN2, COL6A5, SOS1, MET*
Gap junction	-2.43	3E-03	*MAP2K1, HTR2A, ADCY8, GRM1, GNAI2, GRM5, TUBB6, TUBA1A, PLCB4, GNAS, GRB2, SOS1, HRAS, MAP2K5, PRKG1*
Axon guidance	-1.91	2E-02	*NTNG1, SEMA5A, EPHA5, EPHA8, LIMK1, LRRC4, NTN4, UNC5C, L1CAM, GNAI2, EFNB2, RASA1, PLXNB3, FYN, SRGAP2, HRAS, MET*
PI3K-Akt signaling pathway	-1.45	2E-02	*PHLPP1, LAMA2, ATF6B, EPO, RELA, COMP, CCND1, EIF4EBP1, TNR, ITGB8, HRAS, JAK3, MAP2K1, COL27A1, LAMB2, ITGA3, F2R, NFKB1, KITLG, CDK6, COL4A1, COL6A2, COL5A2, COL6A1, DDIT4, ITGA8, COL4A5, MTCP1, GRB2, COL6A6, COL6A5, PKN1, TEK, SOS1, MET*
MAPK signaling pathway	-1.52	3E-02	*PPP5D1, ARRB1, ECSIT, RASGRP1, ELK1, RELA, RASGRP3, DUSP10, GNA12, NTF3, CACNA1S, CD14, HRAS, MAP2K5, MAP2K6, MAP2K3, TGFB2, MAP2K1, MEF2C, DUSP2, MAP3K1, NFKB1, MAPK10, RASA1, GRB2, SOS1, MAP3K14*
Pathways in cancer	-1.38	4E-02	*LAMA2, EPAS1, BRCA2, ADCY8, RASGRP1, RELA, GNAI2, RASGRP3, CCND1, TPR, GNA12, CTNNA1, VHL, CTNNA2, SKP2, HRAS, APC2, TCF7L2, TGFB2, MAP2K1, TCF7L1, CDKN2A, LAMB2, ITGA3, F2R, STAT3, NFKB1, PML, MAPK10, KITLG, PLCB4, CDK6, COL4A1, GNAS, COL4A5, GRB2, SOS1, MET*
Insulin secretion	-1.85	7E-02	*KCNU1, PLCB4, ATF6B, KCNMB2, ATP1A4, GNAS, KCNMB4, KCNN2, CACNA1S, ADCY8, TRPM4*
Cell cycle	-1.61	9E-02	*TGFB2, CDKN2A, BUB1B, TTK, CDC7, SMC1B, CCNA1, CDK6, CCND1, ATM, SKP2, E2F5, BUB1, MAD1L1*

Note: Group #5: Uniquely expressed GC-NR HTM cells specific pathways enriched from DEGs of Group #3 minus Group #2; Size: Number of genes enriched.

Supplementary Table S7: Comparison of Differentially Expressed Genes in TM Cells Treated with Glucocorticoid from Previous Reports and the Present Study

**Study Design**	**Use of Postmortem Tissue**	**Age of the donors (Yrs)**	**Number of Donors used**	**Passage of Cells**	**Dexamethasone Treatment(nM)**	**Duration of Exposure (d)**	**Control medium**	**History of glaucoma**	**Microarray/RNA Seq**	**List of Up-regulated genes**	**References**
Human Cultured TM cells	yes	7,16,21 and 28	4	4-5	100	7	0.1% Ethanol	Not specified	cDNA	*RGC32*, *OCA2*, *ANGPTL7*, *MYOC*, *FKBP5*, *SAA1*, *ZBTB16*	#7
Human Cultured TM cells	Yes	unknown	2	4-6	100	10	0.1% Ethanol	No history of glaucoma	Affymetrix Human Genome U95Av2 gene Chip microarrays	*SERPINA3*, *AKR1C1*, *IGFBP2*, *MYOC*, *PEDF*, *PCOLCE*, *AKR1C3*, *PTGDS*, *CHI3L1*, *ACTG2*, *CDT6*, *ASS*, *CTP*, *APOD*, *AP1G1*	#8
Human Cultured TM cells		unknown		8	100	10	Water in DMEM medium	Not specified	MicroMax Human cDNA System I	*GAS1*, *CDH4*, *MT1L*, *CST3*, *TSC22*, *LDHA*, *IGFBp2*, *TAGLN*	#9
Human Cultured TM cells	Yes	12,16 and 17	3	5	100	21	DMEM medium containing 10% fetal calf serum	No history of glaucoma	Affymetrix Human Genome U133A array	*MYOC*, *DCN*, *IGFBP2*	#10
Human Cultured TM cells	Yes	52	1	8	100	7	0.0025% and 0.025% Benzyl alcohol	No history of glaucoma	Standford Human cDNA SHEW	*GAS1*, *CDH4*, *MT1L*, *CST3*, *ATF4*, *ASNS/TS11*, *CHOP*, *HSPA5*	#11
Human Cultured TM cells	Yes	3 months old and 35	2	3-6	1000	24h	0.1% DMSO	Not specified	Agilent-014850 whole Human Genome Microarray 4X44K G4112F	*RGC32*, *OCA2*, *SAA1*, *ANGPTL7*, *ITGA10*, *LSP1*, *KCNB1*, *FKBP5*, *KLF15*, *ANGPTL4*, *MYOC*, *ZBTB16*, *PF4*	#12
Human Cultured TM cells	Yes	Not specified	3	Not specified	100	14	DMEM with 10% fetal calf serum	Not specified	Agilent -028004 SurePrint G3 Human Gene Expression 8X60 K	*FKBP5*, *ZBTB16*, *SCNN1A*, *SAA1*	#13

									Microarray 039381		
Human Cultured TM cells	Yes	27	1	2	500	6	0.1% ethanol	Not specified	Affymetrix Human Gene 1.0 ST Array	*ITGB3*, *CHN1*, *PIK3R1*, *MFGE8*	#14
Bovine – Perfusion organ culture system -BTM cells with known GC responsiveness used	Yes-Bovine donor eyes	Adult cows	6 donors (paired eyes)	>3	100	7	0.1% ethanol	Not specified	Illumina Human Methylation 450K Chips	*DKK1*, *HMGA2*, *SOD2*, *STEAP4*, *SLC7A8*	#16
Human – Perfusion organ culture system -HTM cells with known GC responsiveness	Yes	67 (mean)	8 donors	3-4	100	7	0.1% Ethanol	No history of glaucoma	Illumina Next Seq 500	*ZBTB16*, *SAA1*, *MYOC*, *OCA2*, *H19*, *APOD*, *SAA2*, *HIF3A*, *PRODH*, *FKBP5*, *LSP1*, *ANGPTL7*, *CPM*. *STEAP4*, *MT2A*, *IRF7*	Present Study

Supplementary Table S8a. Comparison of Pathways Involved in DEX treated TM Cells from Previous Reports and the Present Study

Effect of DEX on TM cells		Present Study
**Human Studies Combined***	**Bovine**[ł]	
Collagen	Collagen	-
ECM	ECM	-
Adhesion	Adhesion	Adhesion
WNT signaling	WNT signaling	WNT signaling
Inflammation	Inflammation	-
Adipogenesis	Adipogenesis	-
Glucose metabolism	Glucose metabolism	-
NF-κB	NF-κB	-
Apoptosis	Apoptosis	-
GPCR	GPCR	-
Oxidative stress	Oxidative stress	-
		Phosphatidylinositol signaling system
		TGF Beta Signaling Pathway
		P53 Signaling Pathway
		mTOR signaling pathway
		Cell cycle
		Adherens junction
		Regulation of Actin Cytoskeleton
		TNF signaling pathway
		MAPK Signaling Pathway
		Pathways in Cancer
		PI3K-Akt signaling pathway
		Cell Adhesion Molecules Cams
		Calcium signaling pathway
		Axon Guidance
		Inflammatory mediator regulation of TRP channels

	ECM-receptor interaction
	cAMP signaling pathway
	Ras signaling pathway
	Steroid biosynthesis
	Vascular smooth muscle contraction
	VEGF Signaling Pathway
	ABC transporters
	Notch signaling pathway
	Gap junction
	cGMP-PKG signaling pathway

Note: [*]*Ref. No.7*-14, [†] Ref.No.16, 35.

Supplementary Table S8b. Comparison of Pathways Enriched in Responder and Non-Responder HTM Cells from Previous Report and the Present Study.

Responder vs. non-responders Bovine ‡	Present Study	
	Responder	Non-Responder
Collagen	-	
ECM	-	
Adhesion	Adhesion	
WNT signaling	WNT signaling	
Inflammation	-	
Adipogenesis	-	
Glucose metabolism	-	
NF-κB	-	
Apoptosis	-	
Cell cycle	Cell cycle	
Senescence	-	
Oxidative stress	-	
	Axon guidance	Long-term depression
	Regulation of actin cytoskeleton	Vascular smooth muscle contraction
	ECM receptor interaction	Inflammatory mediator regulation of TRP channels
	Focal adhesion	Fatty acid metabolism
	PI3K-Akt signaling pathway	VEGF signaling pathway
	Adherens junction	Oxytocin signaling pathway
	Protein processing in endoplasmic reticulum	Calcium signaling pathway
	T cell receptor signaling pathway	ECM-receptor interaction
	Calcium signaling pathway	Focal adhesion
	Cell adhesion molecules (CAMs)	Gap junction
	B cell receptor signaling pathway	Axon guidance

	TNF signaling pathway	PI3K-Akt signaling pathway
	Chemokine signaling pathway	MAPK signaling pathway
		Pathways in cancer
		Insulin secretion
		Cell cycle

Note: ‡ *Ref. No.*16, 35
